# Tumor-Aware Unsupervised Digital HER2 Immunohistochemistry via Dual Contrastive Learning

**DOI:** 10.3390/bios16090524

**Published:** 2026-09-19

**Authors:** Dong-Bum Kim, Jong-Ha Lee

**Affiliations:** Department of Biomedical Engineering, College of Engineering, Keimyung University, Daegu 42601, Republic of Korea; 1006120@stu.kmu.ac.kr

**Keywords:** breast cancer, digital staining, immunohistochemistry, human epidermal growth factor receptor 2, unsupervised learning, contrastive learning

## Abstract

Breast cancer is the most common cancer and the second leading cause of cancer-related death among women worldwide. For accurate diagnosis, pathologists use immunohistochemical (IHC) staining for biomarkers such as human epidermal growth factor receptor 2 (HER2) as an auxiliary test, in addition to hematoxylin and eosin (H&E) staining for evaluating tissue morphology. Traditional IHC staining is limited by high cost, time, and labor, with a shortage of pathologists to meet demand. In this work, we propose a digital HER2 IHC staining algorithm based on tumor masks. The proposed model builds on the Dual Contrastive Learning Generative Adversarial Network (DCLGAN), adding tumor mask loss, structural similarity index measure (SSIM) loss, and a modified identity loss that differentiates between active and inactive regions. Performance was quantitatively assessed by comparing HER2 scores using the Fréchet Inception Distance (FID) and a grade classification model. Compared to DCLGAN, the proposed model achieved a 27.2% decrease in FID and a 2.57% increase in soft accuracy. Visual evaluation further showed that it prevents yellow discoloration and suppresses nonspecific region staining seen in unsupervised learning. These results indicate that effective learning is achievable even with limited and unaligned data, thereby accelerating digital pathology workflows.

## 1. Introduction

Breast cancer is the most common disease among women, accounting for 32% of newly diagnosed cases globally [1]. It poses a serious risk to women’s health and is the second most common cause of cancer-related deaths in women [2]. To diagnose cancer, a pathologist first performs staining on tissue samples obtained through biopsy or surgery [3,4]. Among various tissue staining methods, hematoxylin and eosin (H&E) staining is the most widely used technique for assessing anomalies in tissue morphology [5]. This technique produces contrast between nuclei and extracellular stroma [6]. Eosin stains the cytoplasm pink, whereas hematoxylin mainly stains cell nuclei blue [7]. By emphasizing the morphological characteristics of tissues, this method enables pathologists to visually inspect tissue and cellular structures to make a preliminary diagnosis [4,8]. However, the staining procedure is time-consuming, the interpretation of H&E staining is subjective, and some tasks show a high level of inter-observer variability [9,10,11]. Additionally, auxiliary diagnostic tests like immunohistochemical (IHC) staining are employed when assessing complicated or difficult cases [12].

IHC staining complements the morphological information obtained through H&E staining. Within cellular compartments and suitable histological contexts, it finds and measures the distribution and localization of particular markers [13,14]. This makes it possible to classify tumor subtypes, evaluate prognoses, and choose individualized treatments [13,14]. Positive tumor cells are stained brown and negative tumor cells are stained blue in IHC staining, which is based on the particular binding principle between antigens and antibodies [15]. Human epidermal growth factor receptor 2 (HER2), marker of proliferation Ki-67 (Ki67), estrogen receptor (ER), progesterone receptor (PgR), and cytokeratin 5/6 are five types of IHC markers frequently used in breast cancer [16,17]. The HER2 protein level determines the likelihood of breast cancer cells dividing and replicating [18]. Biomarkers such as HER2, present on the surface of cancer cells, are used to determine the stage of breast cancer [19]. HER2 grading consists of four stages (0, 1+, 2+, 3+), which help determine the appropriate treatment approach should be applied [15,20]. While traditional IHC staining methods are essential for diagnostic accuracy, the staining process is lengthier and more costly [12,21].

**Figure 1:**
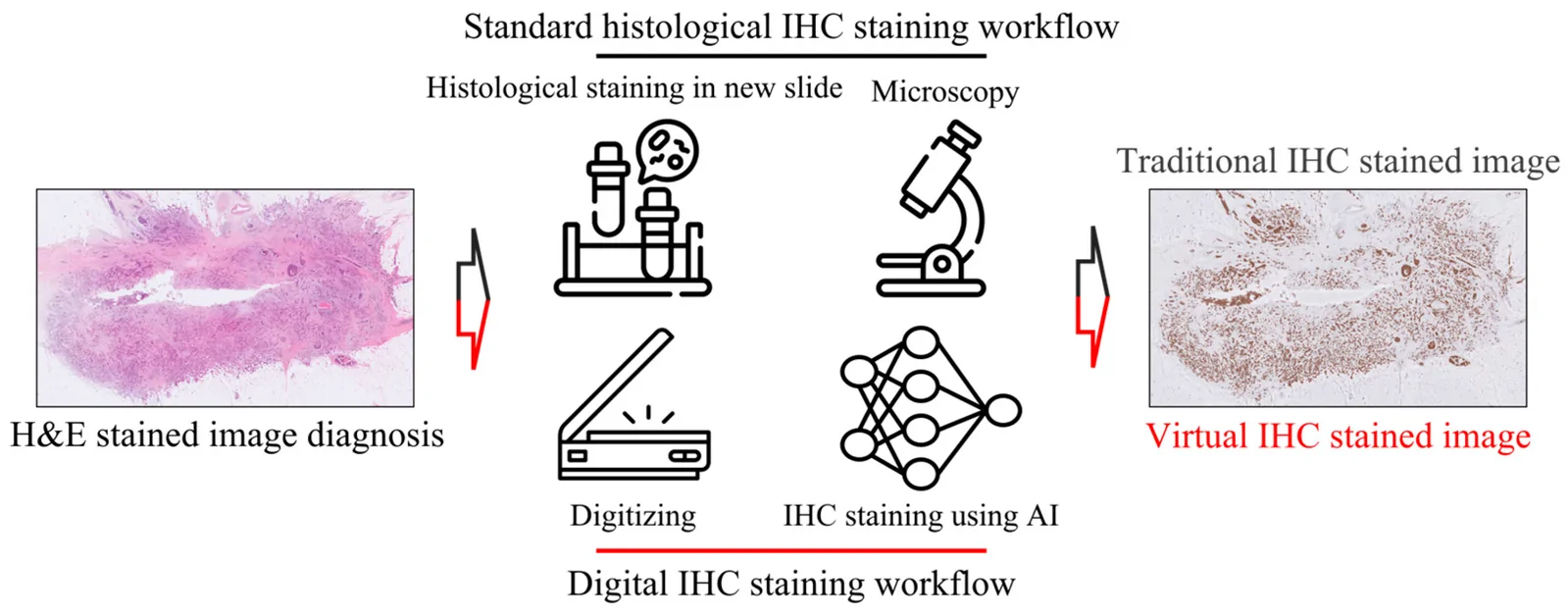
Workflow of the digital IHC staining. The top row shows the conventional standard IHC staining process, where a new slide is prepared when IHC staining is needed, histological IHC staining is performed, and the slide is then observed under a microscope. The bottom row shows the digital IHC staining process, where tissue slides are digitized and virtual IHC staining results are generated through an AI-based model.

Digital staining (DS) technology was created to overcome these difficulties (Figure 1), and more recently, techniques that use Generative Adversarial Networks (GANs) [22] have been investigated to automate this procedure [23]. The biological basis for this transformation lies in the fact that morphological features extracted from H&E images can encode information correlated with underlying molecular and protein expression. Prior studies have shown that deep learning features derived from H&E images statistically correlate with gene expression patterns [24], and that nuclear morphological characteristics (e.g., nuclear size, pleomorphism, nucleolar features) differ significantly between HER2-positive and HER2-negative breast cancer subtypes [25], providing a biological rationale for inferring HER2 expression status from H&E morphology alone. Depending on the kind of data used, DS techniques can be divided into supervised and unsupervised learning approaches. Supervised learning techniques are mainly based on the pixel-level learning framework of Pix2Pix [26] and are usually applied to specialized microscopic data, such as autofluorescence microscopes [27], Fourier ptychographic microscopes [28,29], and holographic microscopes [30]. On the other hand, algorithms based on models like CycleGAN [31], which uses a cycle-consistency approach; Contrastive Unpaired Translation (CUT) [32], which uses contrastive learning; and Dual Contrastive Learning GAN (DCLGAN) [33] are used in unsupervised learning, which primarily uses optical microscopy data.

DCLGAN employs bidirectional contrastive learning between both domains, enabling more robust feature alignment between H&E-stained and IHC-stained representations without requiring pixel-level registration. This property makes DCLGAN particularly suitable for unaligned H&E-stained to IHC-stained virtual staining tasks, which is further supported by the experimental results, where DCLGAN achieved superior performance compared to CycleGAN and CUT.

Recently, methods have been proposed for converting H&E-stained images to specially stained images [34,35] and for converting H&E-stained images to IHC-stained images [36,37] based on the cycle-consistency approach. Additionally, contrastive learning techniques have been used in unsupervised learning-based research to transform H&E-stained images into IHC-stained images [14,38,39]. However, H&E-stained and IHC-stained images frequently show minute spatial differences in cellular positions owing to variations in the staining process. As a result, the two images must be aligned into a relatively registered form. Such registration is typically carried out using image correlation, but this method requires additional alignment steps and can be challenging to implement when the image positions are mismatched or interleaved.

This work proposes a digital HER2 IHC staining algorithm that makes use of a tumor mask to address these limitations. The goal of the proposed method is to transform H&E-stained images into HER2 IHC-stained images. The model is based on DCLGAN, which uses contrastive learning and bidirectional loss. Since HER2 IHC staining selectively stains only specific membrane regions of cells, the domain distribution between H&E-stained and IHC-stained images is fundamentally different, making contrastive learning particularly effective for capturing these domain-specific staining patterns.

Although DCLGAN has shown promising results in unsupervised image translation, it does not account for the domain-specific characteristics of HER2 IHC staining, resulting in undesired staining in nonspecific regions.

**Figure 2:**
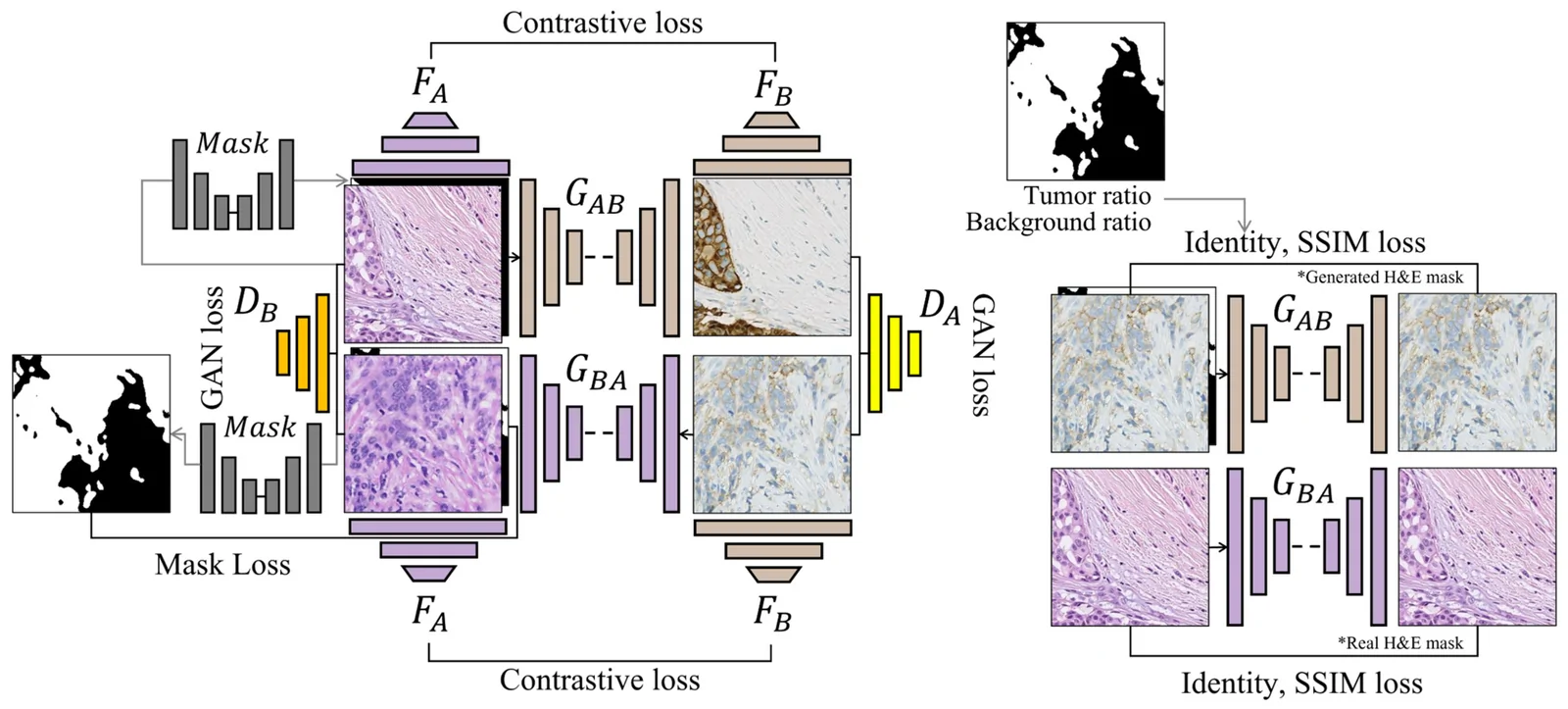
Proposed method. DCLGAN represents a model structure that generates HER2 IHC images utilizing five loss functions (GAN, contrastive, identity, SSIM, and mask loss) and a tumor mask. $G_{AB}$ takes a 4-channel input and outputs a 3-channel image, whereas $G_{BA}$ takes 3-channel input and outputs a 4-channel image. Brown components are associated with HER2 image generation and feature extraction, while purple components are associated with H&E image generation and feature extraction. Yellow and orange represent two separate discriminators. Asterisks (*) indicate the masks used to represent the tumor and background ratios, which were used to calculate the identity loss between the images.

To improve the preservation of tissue structure during the conversion process, the proposed model adds tumor mask loss and structural similarity index measure (SSIM) [40] loss in addition to the losses used in DCLGAN. To reduce needless transformations and improve conversion accuracy, the identity loss is also modified to distinguish between active and inactive regions during training. Specifically, the tumor mask effectively directs the regions of the H&E-stained image that require HER2 IHC staining, while the SSIM loss and modified identity loss maintain tissue structure and ensure that the quality of the converted HER2-stained images resembles the original. The overall workflow of the proposed method is shown in Figure 2.

The main contributions of this study are summarized as follows:

• We propose an extension of DCLGAN for digital IHC staining by incorporating tumor mask and structural loss.
• The proposed network represents a foundational step toward a digital model capable of eventually reducing reliance on conventional histological HER2 IHC staining. If realized in future clinical workflows, this could reduce the time and cost needed to obtain HER2 IHC-stained images and simplify labor-intensive, repetitive procedures.
• By suppressing the IHC staining of nonspecific regions that can occur in an unsupervised learning environment, it prevents the yellow discoloration from appearing across the entire image and reduces unintended staining.
• The proposed model is designed to enable effective learning even in situations with unaligned data and can function without the need for any additional image registration procedures.

## 2. Related Works

Digital IHC staining algorithms have mostly been published using contrastive learning approaches. Numerous studies have developed networks that generate IHC staining, including HER2, Ki67, ER, and PgR from H&E staining.

Li et al. [38] developed the Weakly Pathological Consistency Constraint (WPCC) based on CUT to solve the problem of nonpixel-aligned datasets. Multi-layer embedding vectors are extracted by WPCC from both generated and actual IHC-stained images. The Jensen–Shannon divergence quantifies the distribution difference between the two images, while the Pearson correlation coefficient serves as a weighting factor to determine the degree of alignment. The discriminator also includes a normalized loss function. The Multi-IHC Stain Translation (MIST) [21] and Breast Cancer Immunohistochemical (BCI) [41] datasets were used to train their model. In terms of Peak Signal-to-Noise Ratio (PSNR), FID [42], Kernel Inception Distance (KID), and SSIM metrics, it outperformed CycleGAN, CUT, Pix2Pix, Pyramid Pix2Pix [41], and ASP [21].

Wang et al. [39] proposed a Mix-Domain Contrastive Learning (MDCL) method based on CUT. To estimate correlations using a contrastive loss that took into account both intra-domain and inter-domain similarities, this method used two Siamese networks to extract embedding vectors between anchor and intra/inter-domain patches. Additionally, pixel-wise alignment errors were addressed by applying an adaptive weighting technique. This model was trained on the BCI and MIST datasets, respectively, and showed superior performance in FID, KID, and Perceptual Hash Value (PHV) metrics compared to CycleGAN, CUT, Pix2Pix, Pyramid Pix2Pix, and ASP.

Pati et al. [14] incorporated both local and global consistency losses into a CUT-based framework to assess and train for cell-level accuracy and overall image style consistency. Content and style consistency between the generated IHC image and the original H&E image are enforced by the global consistency loss. The local consistency loss preserves staining and morphological consistency at the cell level by using two distinct networks, a discriminator and a classifier, to predict cellular staining states. In terms of FID and Contrast-Structure Similarity (CSS) metrics, their model outperformed CycleGAN, CUT, CUT+KIN, and AI-FFPE after being trained on the European Multicenter Prostate Cancer Clinical and Translational Research Group (EMPaCT) TMA dataset.

Nevertheless, to correct for imperfect alignment between H&E and IHC images, these three approaches rely on the correlation between the original and generated IHC images. Additionally, they compute global and cell-level consistency losses using multiple patches. However, these methods are limited when there is a large deviation in patch alignment or when it is impossible to establish a perfect one-to-one correspondence between patches.

Asaf et al. [43] proposed a virtual H&E staining method that uses DCLGAN to transform unstained images into H&E-stained images. This approach improved embedding learning performance by combining the bidirectional mapping idea of CycleGAN with the contrastive learning benefits of CUT. Their model outperformed CycleGAN and CUT in FID and KID metrics after being trained on the BioMedical Image and Signal Analysis (BIOMISA) research group dataset.

However, this method has only been studied in the H&E staining transformation task and has not been applied to IHC staining. Therefore, we intend to propose a new approach for IHC staining transformation by extending and applying the DCLGAN-based method in the IHC staining domain.

## 3. Materials and Methods

In this study, we present a digital HER2 IHC staining method that is tumor mask based. The primary objective of this method is to generate HER2 IHC-stained images from H&E-stained images captured by whole slide image scanners (NanoZoomer S360 and NanoZoomer XR, Hamamatsu Photonics K.K., Hamamatsu, Japan), utilizing unaligned data. The traditional DCLGAN algorithm can create HER2 IHC-stained images from H&E-stained images, but this method frequently results in undesired HER2 IHC staining features in nonspecific regions and causes a yellowish discoloration across the entire image. To address this problem, we propose an extended method that combines mask loss, SSIM loss, and modified identity loss based on H&E-stained images.

### 3.1. Preprocessing

Due to the large size of whole slide images (WSIs), five pairs of H&E and HER2 IHC-stained WSIs from the AutomatiC Registration of Breast cancer tissue (ACROBAT) [44] dataset underwent a three-step preprocessing process. The first step involved manually segmenting the entire tissue area from the H&E-stained WSIs and manually extracting the corresponding tissue area from the HER2 IHC-stained WSIs using a bounding box of the same size. To prepare the images for training, the second step involved segmenting them into 256 × 256 patches. Each cropped image’s mean pixel value was determined in the third step, and images with a noticeable background (those with a mean value of 235 or higher) were excluded. For one pair of test WSIs, the entire tissue area was also separated by applying a bounding box, then similarly segmented into 256 × 256 patches, with each image cropped with a 50% overlap.

### 3.2. Algorithm Loss

A tumor masking model was used to extract the tumor mask to identify tumor tissue regions within the H&E-stained images. The H&E-stained image and the extracted tumor mask were then combined to create the input image for the proposed model. By explicitly incorporating tumor region information into the input, the model can focus on learning the staining transformation within biologically relevant regions associated with HER2 expression. Furthermore, by jointly utilizing H&E-stained image information, the model can suppress the generation of undesired staining in nonspecific regions within the tumor regions. In this way, the tumor mask serves as auxiliary biological guidance that is difficult to infer from the H&E image alone, complementing the H&E input rather than replacing it. Thus, H&E staining information and tumor mask information are included in the final input image, which can be expressed as Equation (1):

(1) $$x=\left[x_{rgb}\;;M\left(x_{rgb}\right)\right]$$

Here, $x_{rgb}$ represents the original H&E-stained image, and M represents the tumor masking model. We expressed this relationship as: $M\left(x_{rgb}\right)\approx x_{m}$.

GAN loss function $L_{GAN}$ is based on the GANs algorithm’s adversarial loss [22]. The generator is guided by this loss to generate visually similar images to those in the target domain. In other words, the generated images progressively resemble the original domain through the competitive learning architecture between the discriminator and the generator. In this study, it plays an important role in reducing the domain gap between H&E and IHC images and in generating images that reflect the localized and heterogeneous expression patterns of HER2 staining. Following the least-squares GAN (LSGAN) [45] formulation, which replaces the log-likelihood objective with a least-squares loss to stabilize training, the GAN loss is defined as follow. As a result, the GAN loss can be expressed as Equation (2):

(2) $$\begin{array}{ll} L_{GAN}\left(G,F,\;D_{X}{,D}_{Y},X,Y\right) & \\ & =E_{y\sim Y}\left[{\left(D_{Y}\left(y\right)-1\right)}^{2}\right]+E_{x\sim X}\left[{\left(D_{Y}\left(G\left(x_{rgb}\right)\right)\right)}^{2}\right]\;\\ & +E_{x\sim X}\left[{\left(D_{X}\left(x_{rgb}\right)-1\right)}^{2}\right]+E_{y\sim Y}\left[{\left(D_{X}\left({F\left(y\right)}_{rgb}\right)\right)}^{2}\right] \end{array}$$

Here, the generator G aims to produce images similar to those in domain Y, while the generator F is designed to generate images that resemble those in domain X. The discriminators $D_{X}$ and $D_{Y}$ serve to distinguish between generated images and original images. In this process, generated images are assigned a value of 0 and original images a value of 1, training the model to produce images that closely approximate the originals.

The CUT framework states that the contrastive loss is trained to maximize the mutual information between input and output image patches and is based on the noisy contrastive estimation framework [46,47,48]. During this process, the model learns to differentiate certain patches in the output image from patches at other locations while making them strongly correlated with their corresponding patches in the input image. For instance, HER2 IHC-stained tissue patches with comparable textures should be more closely linked to an H&E-stained image with distinctive textures. As a result, the model is trained to maintain characteristics of the input image in the generated image. A patch taken from the generated image is referred to as the “Query,” the patch at the matching location in the output image is referred to as the “Positive,” and all patches from other positions are referred to as “Negatives” when comparing two image patches [33].

The Query, Positive, and Negative are each converted into Κ dimensional vectors, denoted as v, $v^{+}\in R^{Κ}$, $v^{-}\in R^{N\times Κ}$. Here, N represents the number of negative patches, and $v^{-}\in R^{Κ}$ denotes the n -th negative vector. These vectors undergo L2-normalization and are then framed as an (N+1)-way classification problem, where the model is trained to maximize the probability of selecting the positive vector over the negative vectors [33]. This process is mathematically expressed using the cross-entropy loss [49] as Equation (3):

(3) $$L\left(v,v^{+},v^{-}\right)=-\log\left[\frac{exp\left(sim\left(vv^{+}\right),/,\tau \right)}{exp\left(sim\left(vv^{+}\right),/,\tau \right)+\sum_{n=1}^{N}exp\left(sim\left(vv_{n}^{-}\right),/,\tau \right)}\right]$$

Here, $sim\left(\upsilon ,v\right)=\upsilon^{\tau }v/\left\|\upsilon \right\|\left\|v\right\|$ represents cosine similarity between υ and v. And τ represents a temperature parameter to scale the distance between the query and other patches; we used 0.07 as default.

Patch Noise Contrastive Estimation (PatchNCE) loss function $L_{PatchNCE}$ is used to increase the similarity between real and generated image patches. For HER2 IHC staining, which exhibits localized and heterogeneous expression patterns, such contrastive learning helps preserve important fine-grained patterns, including histological structures and detailed morphological features. For domain X, features are extracted using $G_{enc}$ and $H_{X}$, while the domain Y, features are extracted via $F_{enc}$ and $H_{Y}$. To enhance the diversity and embedding quality across the two distinct domains, we did not share weights between these networks. Specifically, L layers were selected from $G_{enc}\left(x\right)$ and their corresponding features were passed to $H_{X}$ for use in contrastive learning. Also, one image is embedded into a stack of features ${\left\{z_{l}\right\}}_{L}=\;{\left\{H_{X}^{l}\left(G_{enc}^{l}\left(x\right)\right)\right\}}_{L}$. Where $G_{enc}^{l}$ denotes the selected l -th output [33].

In a single image patch, each layer represents distinct features. Utilizing this, we denote the spatial position in each selected layer as $s\in \{1,\ldots ,S_{l}\}$, where $S_{l}$ signifies the number of spatial positions in each layer. Each time, we select one query and denote two corresponding features. The “Positive” is denoted as $z_{l}^{s}\in R^{C_{l}}$, while the remaining “Negatives” are expressed as $z_{l}^{S\setminus s}\in R^{(S_{l}-1)\times C_{l}}$. Here, $C_{l}$ represents the number of channels in each layer. By applying this to domain Y to learn new embeddings, we obtain another stack of features $\{{\hat{z}}_{l}{\}}_{L}={\left\{H_{Y}^{l}\left(F_{enc}^{l}\left(G\left(x\right)\right)\right)\right\}}_{L}$ [33]. The combined forward and backward PatchNCE loss is expressed as Equation (4).

(4) $$\begin{array}{ll} L_{PatchNCE}\left(G,F,H_{X},H_{Y},X,Y\right) & \\ & =E_{x\sim X}\sum_{l=1}^{L}\sum_{s=1}^{S_{l}}L\left({\hat{Z}}_{l}^{s},Z_{l}^{s},Z_{l}^{S\setminus s}\right)+E_{y\sim Y}\sum_{l=1}^{L}\sum_{s=1}^{S_{l}}L\left({\hat{Z}}_{l}^{s},Z_{l}^{s},Z_{l}^{S\setminus s}\right) \end{array}$$

Here, ${\left\{z_{l}\right\}}_{L}=\;{\left\{H_{Y}^{l}\left(F_{enc}^{l}\left(y\right)\right)\right\}}_{L}$ and ${\left\{{\hat{z}}_{l}\right\}}_{L}=\;{\left\{H_{X}^{l}\left(G_{enc}^{l}\left(F\left(y\right)\right)\right)\right\}}_{L}$ are different from G:X→Y.

Identity loss function $L_{IDE}$ [31] prevents the generator’s excessive transformation during the target image generation process. The generated image is encouraged by this loss term to maintain the target image’s unique color and visual qualities. In particular, this loss further helps preserve the staining intensity characteristics of HER2 IHC images, ensuring that biologically meaningful expression patterns are maintained. The tumor mask from the H&E-stained image was used to calculate the ratio of active regions, or tumor and non-tumor areas, prior to calculating the loss [50], as expressed in Equation (5).

(5) $$\alpha =\frac{N}{N+\bar{N}},\;\;\beta =\frac{\bar{N}}{N+\bar{N}}$$

Here, N denotes the total number of pixels in the foreground, which corresponds to the tumor region, and $\bar{N}$ denotes the total number of pixels in the background, which corresponds to the nontumor region. This method helps to differentiate the staining properties of the background and the area of interest.

To reduce the pixel difference between the original image and the output image produced by feeding the original image into the generator of the same domain, we applied an L1 loss. The model gains the ability to maintain its original color and structure without needless modifications through this process. As a result, the identity loss can be expressed as Equation (6):

(6) $$\begin{array}{ll} L_{IDE}\left(G,F,M\right) & \\ & =\alpha_{x}\cdot E_{x\sim X}\left[{\left\|\left(F\left(x_{rgb}\right)⊙M_{x}\right)-\;\left(x_{rgb}⊙M_{x}\right)\right\|}_{1}\right]\\ & +\;\beta_{x}\cdot E_{x\sim X}\left[{\left\|\left(F\left(x_{rgb}\right)⊙\bar{M_{x}}\right)-\;\left(x_{rgb}⊙\bar{M_{x}}\right)\right\|}_{1}\right]\\ & +\alpha_{y}\cdot E_{y\sim Y}\left[{\left\|\left(G\left(\left[y;y_{m}\right]\right)⊙M_{y}\right)-\;\left(y⊙M_{y}\right)\right\|}_{1}\right]\\ & +\beta_{y}\cdot E_{y\sim Y}\left[{\left\|\left(G\left(\left[y;y_{m}\right]\right)⊙\bar{M_{y}}\right)-\;\left(y⊙\bar{M_{y}}\right)\right\|}_{1}\right] \end{array}$$

Here, a meticulous focus is placed on differentiating between the tumor region and the nontumor region. The identity is calculated using only the proportion of each region and the images from the corresponding region. M is a mask activated only for the tumor region, and $\bar{M}$ is a mask activated only for the non-tumor region. Furthermore, it is denoted as $M(F(y{)}_{rgb})\approx y_{m}$.

SSIM loss function $L_{SSIM}$ is used to make sure the generator effectively maintains structural information and visual consistency while creating the target image. This loss reduces distortion in staining intensity and texture and helps the generated image preserve subtle structural patterns from the target image. First, a metric was computed that takes into account the two images’ luminance (l), contrast (c), and structure (s), as defined in Equation (7). And the luminance, contrast, and structure terms are defined in Equations (8)–(10), respectively:

(7) $$SSIM\left(y,\hat{y}\right)={\left[l,\left(y,\hat{y}\right)\right]}^{\alpha }\cdot {\left[c,\left(y,\hat{y}\right)\right]}^{\beta }\cdot {\left[s,\left(y,\hat{y}\right)\right]}^{\gamma }$$

(8) $$l\left(y,\hat{y}\right)=\frac{2\mu_{y}\mu_{\hat{y}}+C_{1}}{{\mu_{y}}^{2}+{\mu_{\hat{y}}}^{2}+C_{1}}$$

(9) $$c\left(y,\hat{y}\right)=\frac{2\sigma_{y}\sigma_{\hat{y}}+C_{2}}{{\sigma_{y}}^{2}+{\sigma_{\hat{y}}}^{2}+C_{2}}$$

(10) $$s\left(y,\hat{y}\right)=\frac{\sigma_{y\hat{y}}+C_{3}}{\sigma_{y}\sigma_{\hat{y}}+C_{3}}$$

Here, $\mu_{y}$ and $\mu_{\hat{y}}$ represent the mean brightness, $\sigma_{y}$ and $\sigma_{\hat{y}}$ represent the standard deviation, and $\sigma_{y\hat{y}}$ represents the covariance.

The coefficients α, β, and γ determine the weight of each component, and $C_{1}$, $C_{2}$, $C_{3}$ are constants added to prevent instability [51]. We use Multi-Scale SSIM (MS-SSIM) [52] as the loss. MS-SSIM is an extended form of the SSIM index that combines measurements at multiple scales of the image to provide greater patch-level robustness against distortion and variations in viewing conditions.

SSIM is used to measure the similarity between the original image and the output image generated by inputting the original into a generator of the same domain. To utilize this similarity value as a loss function, we defined it as 1 minus the SSIM value. That is, a higher SSIM value results in a lower loss, indicating that the generated image is structurally more similar to the original image. Therefore, the SSIM-based loss function is expressed as Equation (11):

(11) $$L_{SSIM}\left(G,F\right)=\left(1-SSIM\left(x_{rgb},\;F\left(x_{rgb}\right)\right)\right)+\left(1-SSIM\left(y,\;G\left(\left[y;y_{m}\right]\right)\right)\right)$$

In this case, the value varies from 0 to 1 based on how similar the two images are, the closer the value is to 0, the more similar the images are, and the closer it is to 1, the more different they are.

The mask loss function $L_{MASK}$ is a loss for the generator to produce a tumor mask similar to the mask generated by the tumor masking model. This loss guides the generator to accurately localize tumor regions, enabling the model to incorporate biologically relevant information and ensuring that staining is applied to tumor regions rather than non-tumor areas. The pixel distance is reduced using L1 loss between the tumor mask generated by the generator and the tumor mask generated using the masking model derived from the H&E-stained image. Therefore, the mask loss function is expressed as Equation (12):

(12) $$L_{MASK}\left(F,M\right)=E_{y\sim Y}\left[{\left\|\;{F\left(y\right)}_{m}-y_{m}\right\|}_{1}\right]$$

Here, $y_{m}$ is the label mask image generated using the tumor masking model, and $F(y{)}_{m}$ is the single-channel mask region of the H&E image produced by the generator.

In summary, the GAN loss guides the generated HER2 IHC-stained images to be visually indistinguishable from real HER2 IHC-stained images, the PatchNCE loss enables the model to learn staining localization according to domain-specific changes, the SSIM loss preserves the structural similarity and visual consistency of the generated HER2 IHC-stained images, the mask loss serves as a guide for concentrating staining in biologically relevant tumor regions associated with HER2 expression, and the modified identity loss stabilizes staining intensity characteristics by distinguishing between tumor and non-tumor regions, further reinforcing the similarity of staining intensity. Finally, the overall generator objective was formulated as Equation (13):

(13) $$\begin{array}{ll} L_{generator}=L_{GAN} & \left(G,F,\;D_{X}D_{Y},X,Y\right)\\ & +\alpha \cdot L_{PatchNCE}\left(G,{F,H}_{X},H_{Y},X,Y\right)\\ & +\beta \cdot \left[L_{IDE}\left(G,F,M\right)+L_{SSIM}\left(G,F\right)+L_{MASK}\left(F,M\right)\right] \end{array}$$

We set α and β to 2 and 1, respectively. Additionally, we used only the GAN loss to compute the discriminator loss.

### 3.3. Architecture

**Figure 3:**
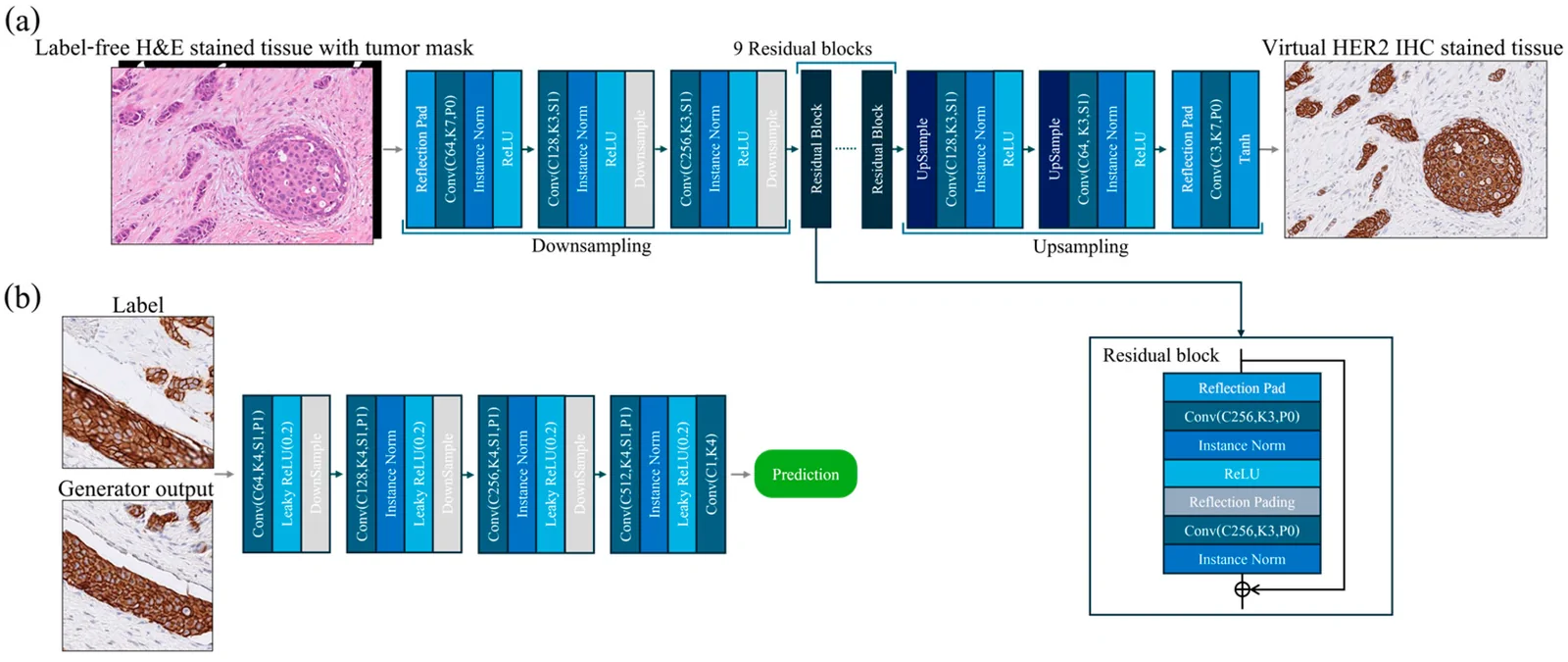
Hierarchical structure of the proposed model (C: Channels, K: Kernel size, P: Padding size). This shows the overall hierarchical structure of the proposed digital HER2 IHC staining model. (**a**) The generator receives the H&E image and its tumor mask as input, and generates a virtual HER2 IHC image through image compression, feature extraction, and image restoration processes. (**b**) The discriminator utilizes a PatchGAN structure to distinguish whether the generated image is an actual original image.

Figure 3 shows the overall hierarchical structure of the proposed digital HER2 IHC staining algorithm. This model transforms images from one domain to another after receiving two domain images (H&E and HER2 IHC) as input. The generator consists of three stages: first, the input image is compressed through anti-aliased downsampling using a blur filter. Next, features are extracted using nine residual blocks based on ResNet [53], following the standard configuration of the official DCLGAN implementation. Finally, anti-aliased upsampling using a blur filter restores the image to its original resolution. The discriminator determines whether the generated image is an actual original image using the PatchGAN structure [41].

The tumor masking model was designed based on the U-Net architecture [54], following the original U-Net configuration. By extending the basic U-Net structure that generates a single mask output, this study modified the structure to generate five types of masks included in the Breast Cancer Semantic Segmentation (BCSS) dataset [55].

The BCSS dataset was used for model training and validation using an 80:20 split, and four data augmentation techniques were applied to ensure data diversity and prevent overfitting. First, random up-down and left-right flips, along with 0°, 90°, and 180° rotations, were performed on the input images. Second, color jitter was applied to adjust brightness, contrast, saturation, and hue within ranges of 0.1, 0.1, 0.1, and 0.05, respectively. Third, Gaussian blur was applied with a kernel size of 3 and a standard deviation σ∈(0.1, 2.0). Finally, normalization with a mean and standard deviation of (0.5, 0.5, 0.5) was applied to all images. The tumor masking model trained in this way was applied to the domain X images of the proposed method, extracting tumor masks from the input images $x_{rgb}$.

### 3.4. Postprocessing

Following a postprocessing step, the generated and original HER2 IHC images were compared using FID and HER2 score. The generated images during the experiment demonstrated a propensity for relatively abnormal patterns in the outer regions, and it was confirmed that the characteristics varied significantly from image to image, which made quantitative comparison challenging. We applied a linear stitching technique during image merging to solve this issue, and we weighted the images in a way that gradually reduced the weight from the center to the periphery. Ultimately, every patch was combined and stored as a single whole color image, which served as the basis for the quantitative assessment.

### 3.5. HER2 Grade Classification Model

We created a HER2 IHC grade classification model and compared HER2 score accuracy to quantitatively assess the HER2 IHC staining quality across models.

Using HER2 IHC-stained images, the HER2 grade classification algorithm categorizes into four grades. We compared seven classification models (ConvNeXt-Base, ViT-Base, EfficientNet-B2, DenseNet-121, Inception-V3, MobileNet-V2, ResNet-50V2) to identify the best-performing architecture for this task. To improve image diversity, we employed augmentation techniques on the BCI dataset. Initially, we applied random transformations such as left-right and up-down flips, brightness adjustments up to 0.1, and contrast adjustments between 0.9 and 1.1. The preprocessing techniques of each corresponding pretrained model were then applied to the data. The classification head was eliminated, and the pretrained models were only utilized for feature extraction. The extracted features were then linked to a fresh multi-layer perceptron (MLP). This classifier’s first layer had a dropout of 0.3 and was stacked with fully connected layers made up of 1024, 512, 256, and 4 neurons. Each layer was followed by batch normalization. Class probabilities were computed in the final output layer using the Softmax function. The weights of the pretrained models were frozen and not trained, and only the new classifier was trained.

To perform a quantitative evaluation among the models, the images used as input were those that had undergone postprocessing. These images were cropped into nonoverlapping 256 × 256 patches, and images at corresponding positions were compared. The comparison was done under the assumption that the location of the original HER2 IHC-stained image and the generated HER2 IHC-stained image were approximately the same. Equation (14) was the basis for the ordinal regression used to score the staining accuracy. It was important to accurately and consistently reflect the relative differences in staining patterns across grades. In issues like the HER2 grade, it is crucial to discourage the misclassification of a patient who is severely ill (score 3+) as healthy (score 0) in comparison to classifying them as score 2+, even though independent classes are not sensitive to order from the model’s training perspective. Furthermore, a weaker penalty was applied for distinguishing between score 3+ and score 2+, as this distinction can vary between human observers and is not perfectly precise due to inherent ambiguities [56]. Therefore, this metric was used solely to evaluate the relative consistency of staining patterns across grades and did not represent a clinical or diagnostic performance measure, which is defined as Equation (14):

(14) $$Soft\;accuracy\left(y,\hat{y}\right)=\left\{\begin{array}{c} 1,\;\;if\;y=\hat{y}\;\;\;\;\;\;\;\;\;\\ 0.5,\;\;if\left|y-\hat{y}\right|=1\;\;\;\\ 0,\;\;if\left|y-\hat{y}\right|\geq 2 \end{array}\right.$$

Here, y is the grade result from the original HER2 IHC-stained image, and $\hat{y}$ is the grade result from the generated HER2 IHC-stained image.

## 4. Results

### 4.1. Datasets

Tumor masking, IHC staining, and grade classification models were trained and evaluated using three publicly available challenge datasets.

The tumor masking algorithm was trained based on the BCSS dataset [55]. This dataset was publicly released in the BCSS Challenge and consists of WSIs acquired from a total of 151 breast cancer tissue samples. It includes 138 WSIs at 40× magnification and 13 at 20× magnification, which were manually annotated by pathologists, pathology residents, and medical students across 22 tissue classes. From the WSIs, we extracted the Region of Interest (ROI), which varied in size from a minimum of 1036 × 1222 pixels to a maximum of 6813 × 7360 pixels. Out of the 22 annotated classes, we used four: tumor, stroma, inflammatory, and necrosis. The other classes were incorporated into other categories. There was an imbalance in the data distribution for each class; the distribution was tumor 40%, stroma 37%, inflammatory 10%, necrosis 6%, and others 7%, but no additional adjustment was made for class imbalance. ROI images were first cropped into 1024 × 1024 patches and resized to 256 × 256 pixels for model training, utilizing a total of 1007 training images and 340 validation images.

The digital HER2 IHC staining algorithm was trained using the ACROBAT challenge dataset [44]. Each sample in this dataset includes one H&E-stained WSI and one to four matched IHC-stained WSIs (HER2, Ki67, ER, PgR) from patients with breast cancer at a 10× magnification. Five WSI pairs were used in this study, four of which were used for training and one for testing. The data were separated at the WSI level, ensuring that patches from the same WSI were not shared across training and testing sets. This setup allows evaluation on an unseen WSI, partially mitigating concerns regarding data leakage and overfitting. Following preprocessing, 10,240 images made up the test data, while 27,201 (H&E) and 29,179 (HER2 IHC) image patches were used as training data.

Ultimately, the BCI dataset [41] was used for the grade classification algorithm that compared and assessed the generated HER2 IHC images with the original HER2 IHC images. Annotated into four grades based on staining intensity, this dataset comprises 4873 pairs of H&E and HER2 IHC images. The resolution of each image is 1024 × 1024 pixels, and the images were divided into 256 × 256 patches for training. A total of 3896 pairs of samples were used for training, and the remaining 977 pairs were used for validation.

### 4.2. Implementation Details

The digital HER2 IHC staining model, the tumor masking model, and the HER2 grade classification model differ in scale and computational requirements. As a result, various experimental settings were used to develop the models. The training progress was verified using the training dataset, and the final performance was assessed using the test dataset because the HER2 IHC staining model has a slower training speed because of the comparatively large amount of data used. By contrast, the training progress was tracked using both the training and validation datasets for the HER2 grade classification model and the tumor masking model, both of which have faster training speeds. Based on empirical settings, learning-related hyperparameters (such as learning rate, batch size, etc.) were modified according to each model’s structure and dataset properties.

A U-Net architecture configuration served as the basis for training the tumor masking algorithm. For training, the learning rate, batch size, and number of epochs were set to 1.0 × 10^−4^, 8, and 100, respectively, using the Adam optimizer [57]. The BCE loss was computed for each individual mask and then averaged to assess the loss for multiple masks. Experiments were carried out on a workstation with a single NVIDIA GeForce RTX 4070 GPU using the PyTorch framework (version 2.7.1).

The DCLGAN configuration was the primary method used to train the digital HER2 IHC algorithm and the same training configuration was applied to all compared models (CycleGAN, CUT, and DCLGAN) to ensure a fair comparison. Hyperparameters β1 = 0.5 and β2 = 0.999 were used to train the Adam optimizer. The learning rate, batch size, and number of epochs were set to 2.0 × 10^−4^, 4, and 30, respectively. After ten epochs, a linear decay of the learning rate was applied. Lastly, the LSGAN loss was used as the GAN loss, and instance normalization [58] was applied. Every experiment was carried out on a workstation with four NVIDIA TITAN Xp GPUs using the PyTorch framework (version 1.6.0). The detailed hyperparameters and experimental settings are summarized in Table 1.

The grade classification algorithm classified five masks (tumor, stroma, inflammatory, necrosis, and others) from the BCSS dataset, with the last class, others, consisting of the masks excluding the first four. The Adam optimizer was used with a learning rate, batch size, and number of epochs set to 1.0 × 10^−3^, 32, and 50, respectively. Every ten epochs, the learning rate was multiplied by 0.9. Every experiment was conducted on a workstation with a single NVIDIA GeForce RTX 3090 Ti GPU using the TensorFlow framework (version 2.10.1).

### 4.3. Model Verification

#### 4.3.1. Analysis of Tumor Masking Model Performance

To evaluate the performance of the tumor masking model, we analyzed the loss and Mean Intersection over Union (mIoU) for training and validation. The mIoU was calculated by averaging of the Intersection over Union (IoU) for each class, as defined in Equation (15):

(15) $$mIoU=\frac{1}{C}\sum_{C=1}^{C}\frac{{TP}_{C}}{{TP}_{C}+{FP}_{C}+{FN}_{C}}$$

where C is the number of classes, True Positive (${TP}_{C}$) denotes the count of cell nuclei pixels measured in the generated H&E-stained image relative to those in the original H&E-stained image. False Positive (${FP}_{C}$) denotes the count of cell nuclei pixels quantified in the generated H&E-stained image relative to the background pixels measured in the original H&E-stained image. Lastly, False Negative (${FN}_{C}$) denotes the count of background pixels measured in the generated H&E-stained image relative to the background pixels identified in the original H&E-stained image [57,59].

**Figure 4:**
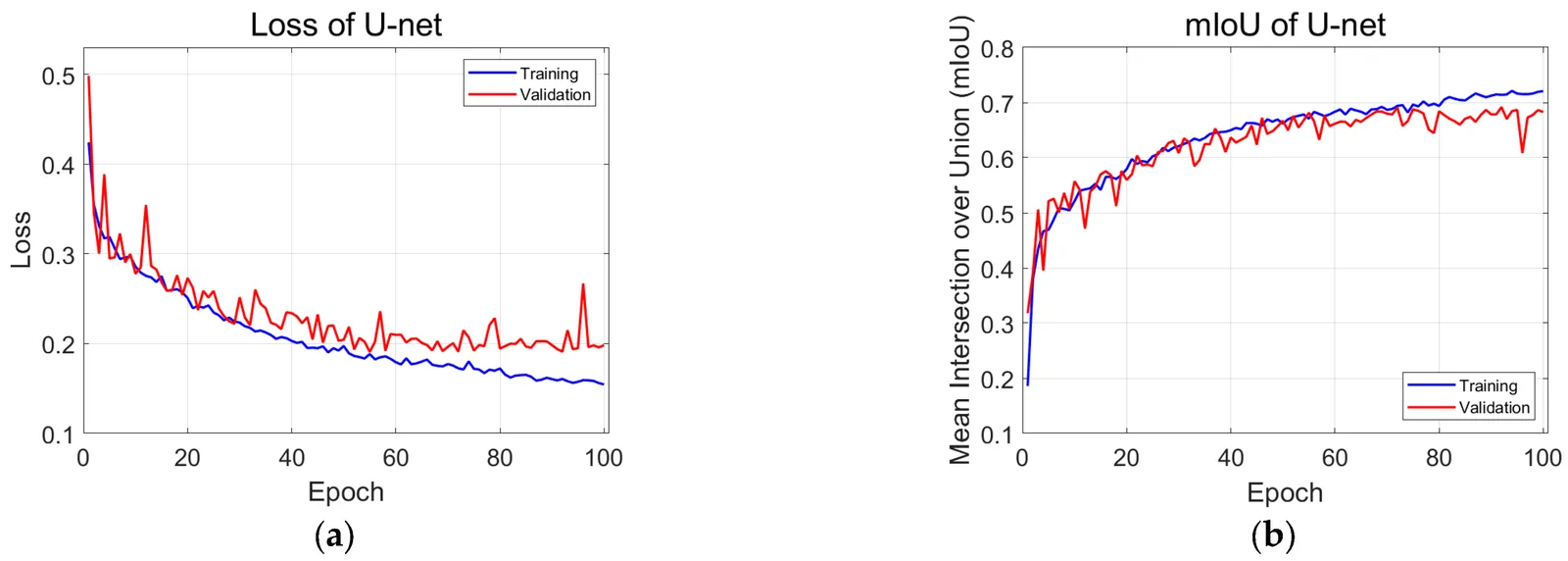
Loss and mIoU curve of tumor masking model. (**a**) Loss of U-Net. (**b**) mIoU of U-Net.

To evaluate the performance of the proposed tumor masking algorithm, we monitored the loss and mIoU values for training and validation data during the training process (Figure 4).

The training loss ranged between 4.24 × 10^−1^ (maximum) and 1.54 × 10^−1^ (minimum), with mIoU values ranging between 7.21 × 10^−1^ and 1.86 × 10^−1^. The validation loss ranged between 4.98 × 10^−1^ and 1.9 × 10^−1^, and the mIoU ranged between 6.91 × 10^−1^ and 3.18 × 10^−1^.

**Figure 5:**
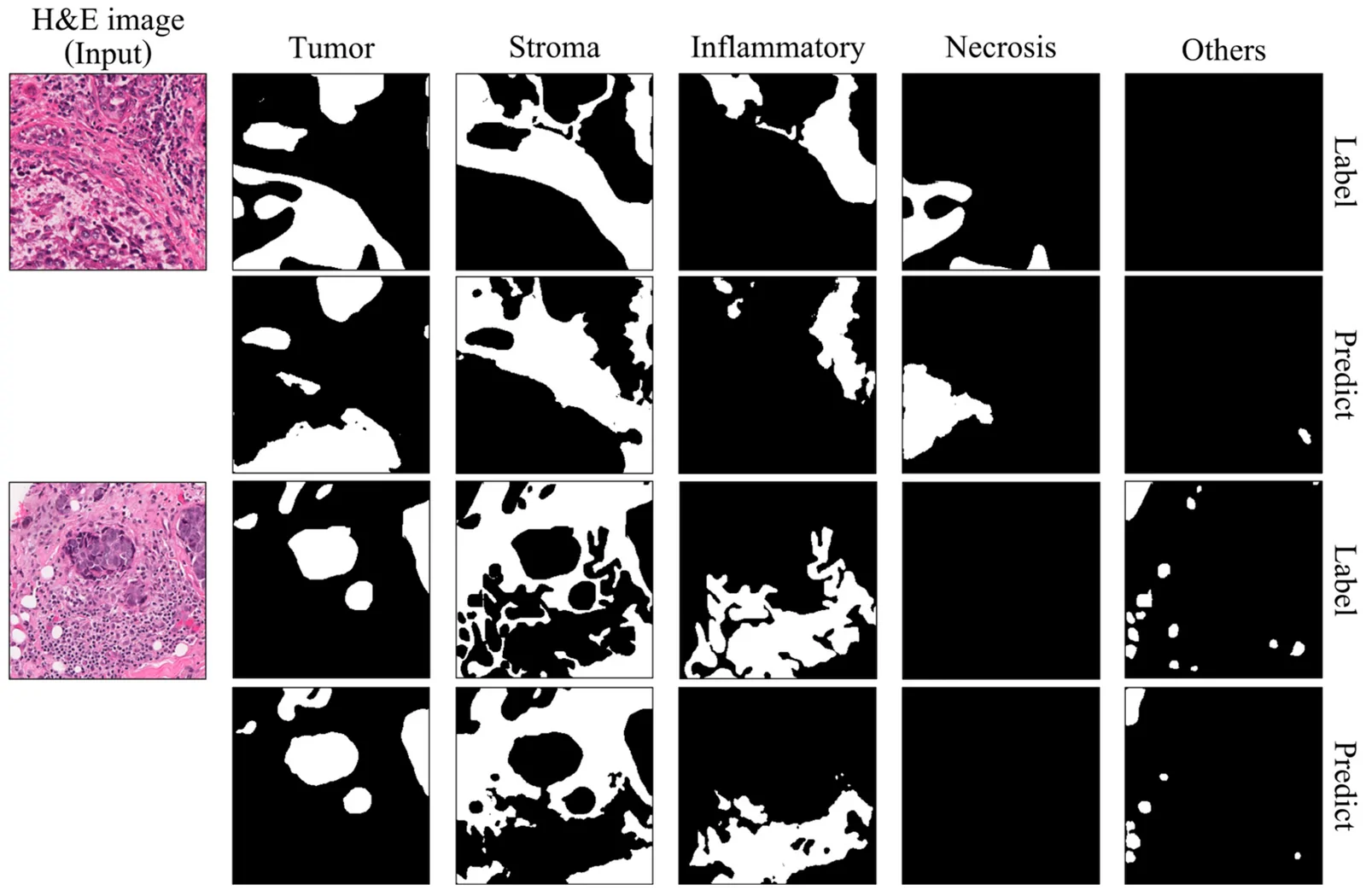
Multi-class segmentation predictions for five tissue classes (tumor, stroma, inflammatory, necrosis, others). For each of the two H&E input samples, the ground-truth label and the model’s prediction are shown. The model identifies main features but shows some deficiencies in fine boundaries and small structures.

Figure 5 shows the validation result images at the point where the mIoU reached its highest value on the validation data. It can be confirmed that the model identifies the main features among the five mask regions effectively. However, it is evident that some details are lacking in fine boundaries or small structures.

#### 4.3.2. Analysis of Digital HER2 IHC Staining Model Performance

**Figure 6:**
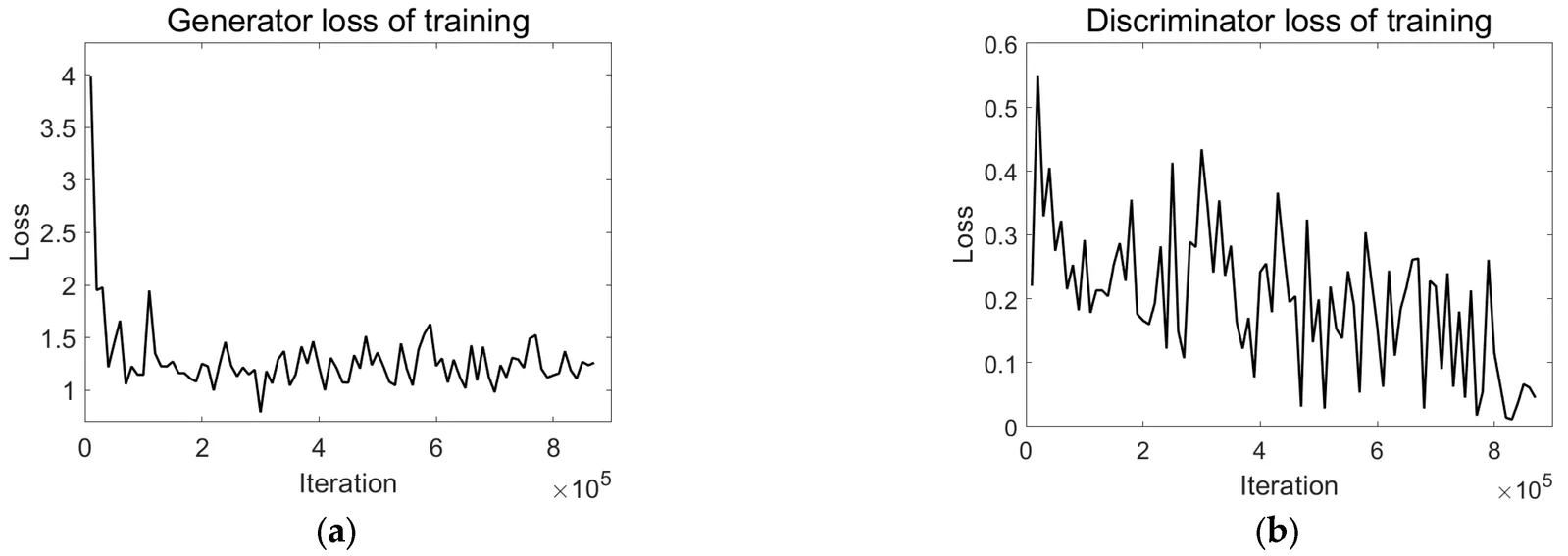
Loss curve of the proposed digital HER2 IHC staining model. (**a**) Generator loss. (**b**) Discriminator loss.

The generator and discriminator loss values were tracked during training to assess the proposed digital HER2 IHC staining algorithm. Specifically, the loss values were recorded every 10,000 iterations to quantitatively analyze the training progress (Figure 6).

The generator loss ranged between 3.98 (maximum) and 7.89 × 10^−1^ (minimum), while the discriminator loss ranged between 5.5 × 10^−1^ and 1.1 × 10^−2^.

#### 4.3.3. Analysis of HER2 Grade Classification Model Performance

**Figure 7:**
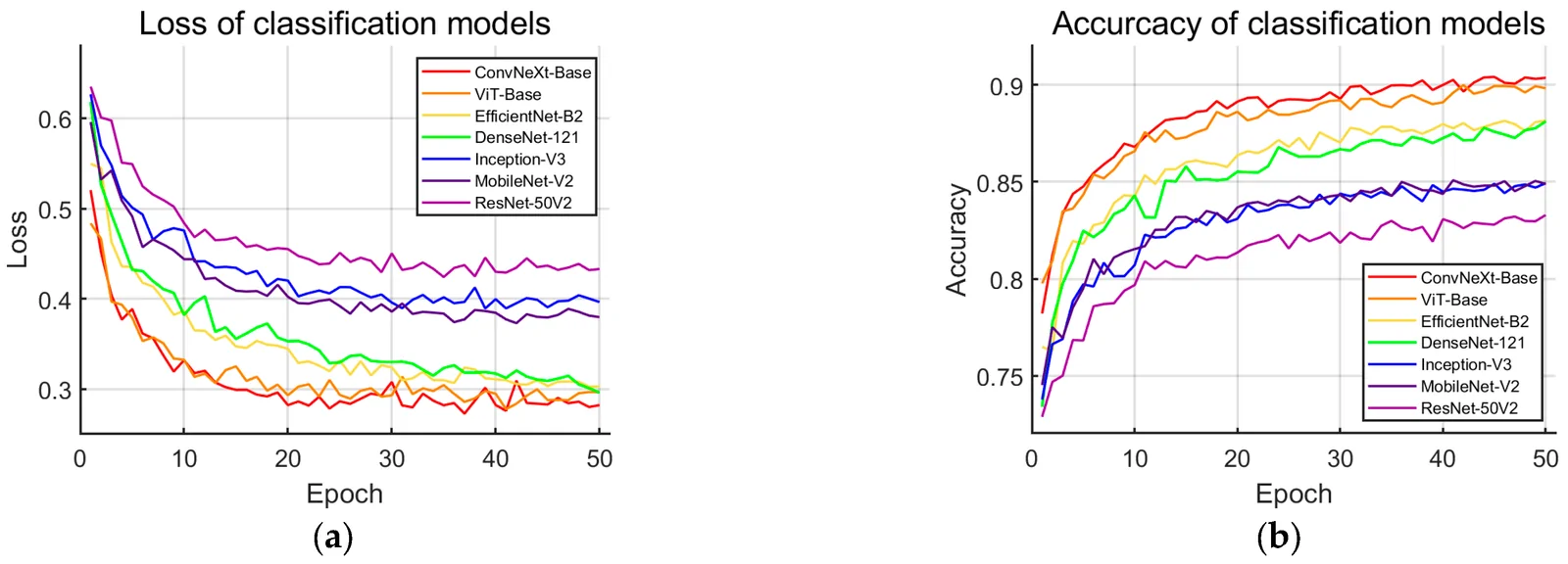
Loss and accuracy curves of HER2 grade classification models. (**a**) Validation loss. (**b**) Validation accuracy.

Finally, the accuracy and validation loss of the seven models were tracked during training to assess the proposed grade classification algorithm. (Figure 7).

The comparison of the maximum values among the validation accuracies of each model is shown in Table 2.

**Table 2:** Performance comparison of seven HER2 grade classification models on the validation dataset. All models trained using identical settings.

Model	Accuracy	F1-Score	Params (M)
ConvNeXt-Base	0.9036	0.8888	89.28
ViT-Base	0.8983	0.8853	87.25
EfficientNet-B2	0.8815	0.8815	9.88
DenseNet-121	0.8812	0.8810	8.75
Inception-V3	0.8493	0.8195	24.57
MobileNet-V2	0.8489	0.8183	4.23
ResNet-50V2	0.8329	0.8013	26.33

Among the compared models, ConvNeXt-Base achieved the best performance with an accuracy of 0.9036 and F1-score of 0.8888. ViT-Base followed with an accuracy of 0.8983 and F1-score of 0.8853. By contrast, ResNet-50V2 showed the lowest accuracy of 0.8329 and F1-score of 0.8013. In terms of model size, ConvNeXt-Base used the largest number of parameters (89.28 M), whereas MobileNet-V2 was the smallest (4.23 M).

**Figure 8:**
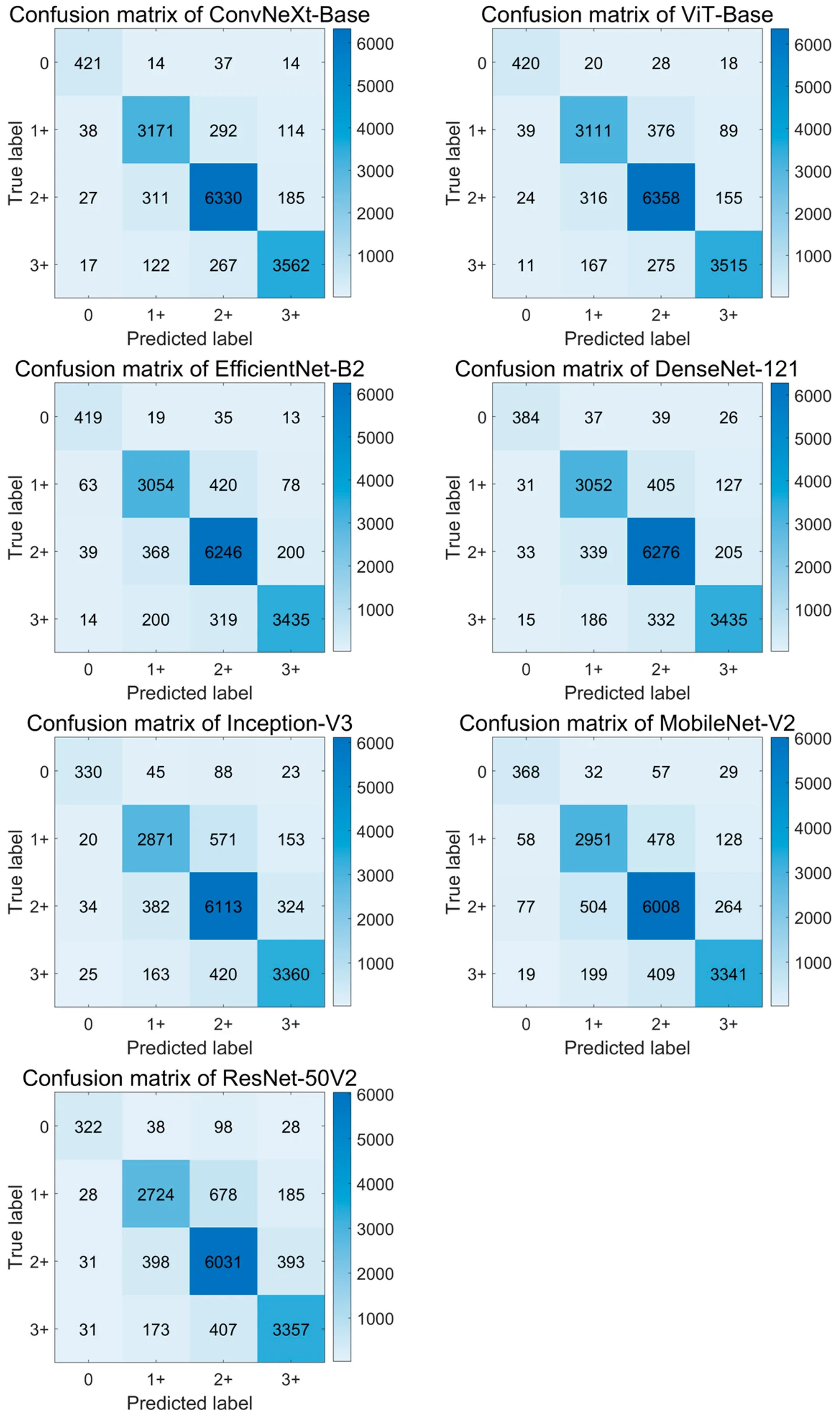
Confusion matrices of HER2 grade classification models with different pretrained models. Each matrix shows the predicted versus true HER2 grades on the validation dataset.

In addition, we created a confusion matrix by contrasting the actual labels with the output of the best-performing models. We examined each class’s prediction performance using the confusion matrix, and the results are displayed in Figure 8. An overall balanced diagonal pattern was found when comparing confusion matrices from various models. The ViT-Base model most accurately predicted 6358 samples in class 2+, whereas the ConvNeXt-Base model showed the best prediction performance for classes 0, 1+, and 3+, with 421, 3171, and 3562 samples correctly predicted, respectively.

#### 4.3.4. Qualitative and Quantitative Comparison of Digital HER2 IHC Staining Models

To verify the validity of the proposed digital HER2 IHC staining, qualitative and quantitative evaluations were performed on the generated HER2 IHC-stained images. The qualitative evaluation was conducted visually, while the quantitative evaluation compared HER2 scores using FID and a grade classification model that substitutes for a pathologist. After postprocessing, the images were cropped to 256 × 256 pixels, and the original HER2 IHC-stained images at the same locations were compared with the generated HER2 IHC-stained images from each model to assess overall similarity.

FID shows a high degree of agreement with human perception, estimating the distribution of original and generated images in the network feature space and calculating the divergence between the two images using the Fréchet distance. The feature vectors for FID are extracted from a pretrained Inception-V3 model. Lower FID values indicate a higher degree of similarity between the two image distributions. The FID is defined as Equation (16):

(16) $$FID={\left\|\mu_{y}-\mu_{\hat{y}}\right\|}_{2}^{2}+Tr\left(\Sigma_{y}+\Sigma_{\hat{y}}-2{\left(\Sigma_{y}\Sigma_{\hat{y}}\right)}^{1/2}\right)$$

Here, the FID score is calculated by computing the Euclidean distance between the means of the feature vectors and adding the trace (Tr) of the combination of the covariance matrices ($\Sigma_{y}$ and $\Sigma_{\hat{y}}$).

In addition, we used the KID as a complementary metric. It uses a polynomial kernel on Inception features and is known to be more reliable than FID for small sample sizes. Following common practice, KID values are reported after multiplication by 100 for better readability.

We further report macro-averaged F1-score and weighted F1-score, computed between the reference and generated HER2 grades using the grade classification model. F1-score is the harmonic mean of precision and recall, providing a balance between false positives and false negatives for each grade. Macro-averaged F1-score computes the F1-score for each grade independently and averages them with equal weight regardless of class size, making it sensitive to performance on underrepresented grades. Weighted F1-score instead averages per-class F1-scores in proportion to each grade’s prevalence in the reference data, reflecting overall agreement under the actual class distribution.

**Figure 9:**
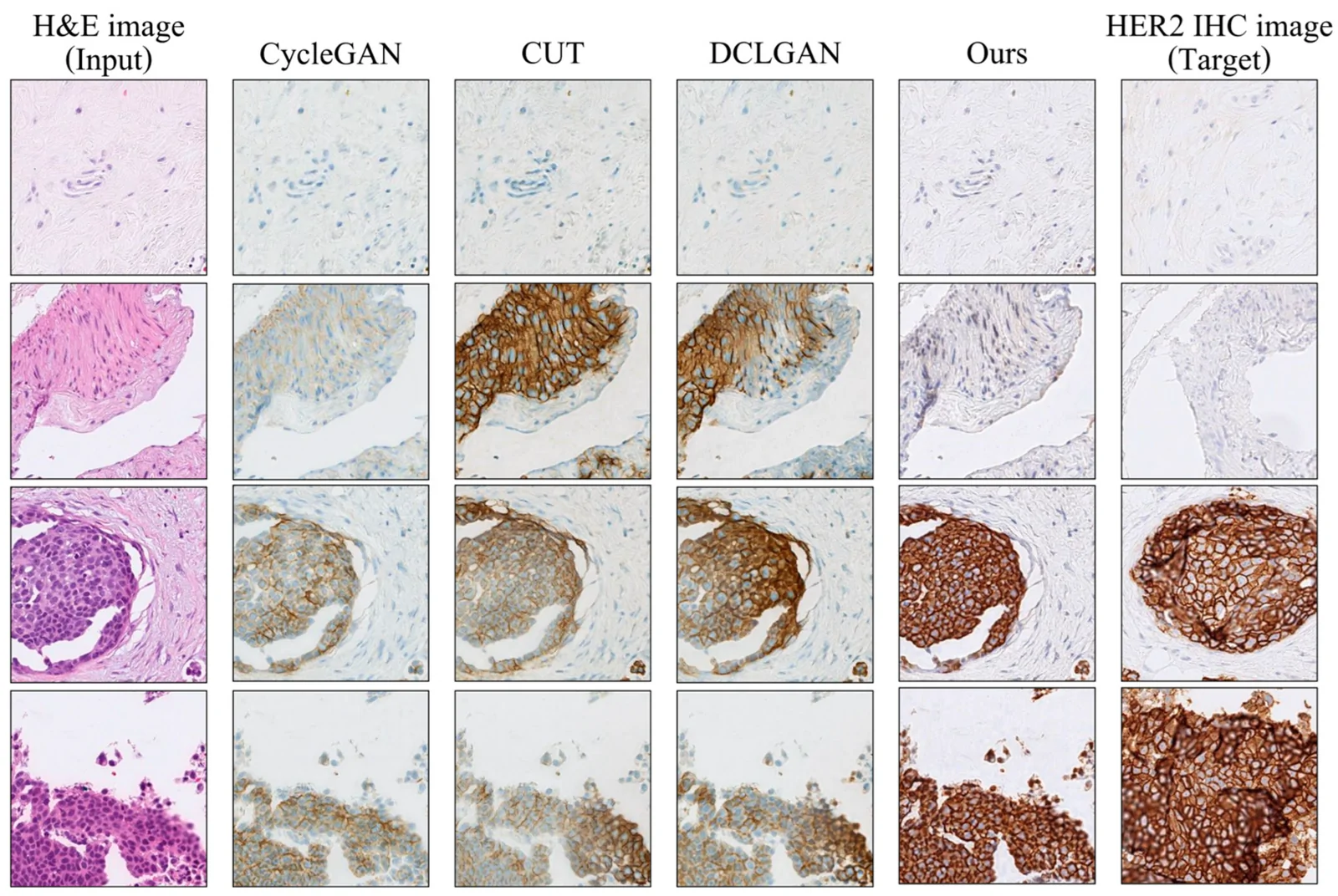
Digital HER2 IHC staining results between models. The virtual HER2 IHC images were evaluated for consistency in staining quality, intensity, and background reproduction compared to the original HER2 IHC images.

The digital HER2 IHC staining model was trained based on four GANs models (CycleGAN, CUT, DCLGAN, Ours). Figure 9 shows examples of the test data demonstrating the performance of each model against the original HER2 IHC-stained.

We visually examined the same locations in the final images for qualitative evaluation. Even in nearby patch images, the HER2 IHC-stained images generated by the proposed model exhibit consistent staining quality, with comparable staining intensity and contrast. However, even after postprocessing, the other models displayed inconsistent staining intensity within a single image. Additionally, while the other models displayed an overall yellow discoloration (the staining color of HER2 IHC expression), the proposed model reproduced a white background in the image, similar to the original HER2 IHC staining results.

Furthermore, this study did not employ supervised learning GAN models like Pix2Pix. Supervised learning requires paired data for training, even though it yields good results. However, in H&E staining and IHC staining, the tissue is deformed during the staining process, and the position is slightly shifted. Because of this, it is challenging to obtain the aligned H&E and IHC-stained images needed for supervised learning. Since this dataset undergoes two separate staining procedures, the tissue deformation also appears differently. Therefore, even relatively aligned data cannot be guaranteed to be correct. Consequently, we only compared unsupervised learning-based models.

We then examined the similarity of FID and HER2 scores to conduct a quantitative assessment. The two images are more similar if the FID score is closer to 0, and the HER2 score is closer to 1. The accuracy of the HER2 score was compared using soft accuracy, which applies weak penalties to adjacent grades. The comparison of the test results of each digital HER2 IHC staining model is shown in Table 3.

CycleGAN has the highest FID of 99.1 and the lowest soft accuracy of 57.33%, showing that image quality and classification consistency are relatively poor. CUT and DCLGAN show mid-level performance with FID scores of 65.7 and 56.6 and soft accuracies of 76.63% and 78.71%, respectively. In contrast, the proposed model has the lowest FID of 41.2 and the highest soft accuracy of 80.73%. As a complementary metric to FID, the proposed model achieved the lowest KID of 1.76, followed by DCLGAN of 2.91, CUT of 3.48, and CycleGAN of 6.56. Regarding F1-based metrics, the proposed model achieved the highest weighted F1-score of 0.593, followed by DCLGAN of 0.591, CUT of 0.533, and CycleGAN of 0.445. For macro-averaged F1-score, DCLGAN achieved the highest value of 0.28, followed by the proposed model of 0.27, CUT of 0.25, and CycleGAN of 0.22.

#### 4.3.5. Ablation Study

To evaluate the individual contribution of each proposed component, we conducted an ablation study starting from the DCLGAN baseline and progressively adding the tumor mask, mask loss, MS-SSIM loss, and modified identity loss. As shown in Table 4, the DCLGAN baseline achieved an FID of 56.6, a KID of 2.91, a soft accuracy of 78.71%, a weighted F1-score of 0.591, and a macro-averaged F1-score of 0.284. Adding the tumor mask alone slightly increased the FID to 58.73 and decreased the weighted F1-score to 0.561, while improving the soft accuracy to 79.67% and the macro-averaged F1-score to 0.303, its highest value across all configurations. Incorporating the mask loss reduced both FID and KID to 57.19 and 2.78, respectively, while the soft accuracy decreased to 77.73% and the weighted F1-score partially recovered to 0.571. Further adding the MS-SSIM loss reduced the FID to 52.21 and the KID to 2.31, with the weighted F1-score improving to 0.586, whereas the soft accuracy remained comparatively low at 77.00%. The full model (Ours), which additionally includes the modified identity loss, achieved the lowest FID of 41.2, the lowest KID of 1.76, the highest soft accuracy of 80.73%, and the highest weighted F1-score of 0.593, while showing the lowest macro-averaged F1-score (0.27) among all configurations.

## 5. Discussion

Many studies on unsupervised digital IHC staining are categorized as unsupervised learning approaches; however, they often implicitly assume that source and target images are relatively aligned or registered during training. In real-world pathological settings, such alignment assumptions are difficult to satisfy consistently due to variations in scanner devices, image acquisition conditions, and tissue sectioning. Also, in unsupervised learning settings without alignment method, the model lacks explicit tissue-specific information. At the result, existing approaches may suffer from nonspecific background staining or color bias in the generated images.

To address these practical limitations, this study designed a digital HER2 IHC staining method that is tumor mask based. Our proposed method consistently improved performance in both HER2 grade classification and FID. These findings demonstrate the strong potential of the proposed system for high similarity to conventional HER2 IHC staining technique (in terms of visual quality and structural similarity).

Despite the promising results, several limitations and challenges remain in this study. The number of WSI pairs used in this study is limited. This is mainly due to the relatively sparse distribution of HER2-positive regions within WSIs. Simply increasing the number of WSIs may introduce a large proportion of non-informative background regions (e.g., normal tissue and background), which can bias the model toward weak staining intensity or widespread nonspecific staining. Although patch-based training increases the number of samples, patient-level diversity remains limited, which may affect the generalizability of the model when applied to data acquired under conditions different from those of the ACROBAT dataset. For example, if H&E images acquired under different staining protocols are used as input, mismatches in staining intensity may occur in the generated HER2 IHC images. Therefore, the model may partially adapt to dataset-specific characteristics, and further validation on larger and more diverse datasets is required to confirm its generalization capability. Although the model is trained using unaligned data, the evaluation is performed by assuming approximate spatial correspondence between patches in order to compare staining presence and intensity. The evaluation reflects overall image-level characteristics; however, it does not rely on precise spatial alignment. Therefore, the results should be interpreted with caution, particularly for fine-grained spatial accuracy. Furthermore, the HER2 classification model was used as a proxy for evaluation and does not represent a clinical or diagnostic performance measure. Since expert pathological assessment was also not available, the results should be interpreted with caution. In addition, the reference HER2 grades used for both training the classification model and evaluating the generated staining outputs are derived from routine clinical IHC assessment, which is known to exhibit substantial inter-observer variability, particularly at grade boundaries (e.g., 2+/3+). Consequently, part of the disagreement reflected in the reported concordance metrics may originate from variability in the reference standard itself, rather than solely from errors in the generated staining. We acknowledge that this study evaluates concordance against the real IHC image and does not independently validate the real IHC image itself against ground-truth HER2 protein expression. Consequently, our results indicate the degree to which the generated staining reproduces the real IHC image, rather than establishing which method more accurately reflects the true underlying HER2 expression level. Comparison against an independent molecular gold standard, such as proteomic quantification of HER2 receptor concentration (e.g., expressed per cell or per milligram of tissue), is considered an important direction for future validation. Through such validation, we also aim to empirically demonstrate the potential reduction in time and labor associated with HER2 IHC staining, which has not yet been established in the present study.

From a practical perspective, the computational complexity of the proposed method is also considered. The proposed method introduces additional components, such as tumor mask input and structural losses, which slightly increase the training cost compared to the baseline DCLGAN. However, since the overall network architecture remains unchanged, the increase in computational complexity is marginal, and the inference cost is largely unaffected.

### 5.1. Performance Analysis of Individual Methods

In the tumor masking experiment, the proposed model showed a tendency for both loss and mIoU to converge stably on the training and validation data (Figure 4). This indicates that the model effectively learned tumor regions and reliably captured the features of masking targets. These findings suggest stable and consistent learning behavior at the patch level during training.

The generator loss in the digital HER2 IHC staining experiment also showed a steady convergence trend (Figure 6a), indicating that the model was successful in producing visually similar images to those in the original domain. The discriminator loss, on the other hand, demonstrated a steadily declining trend as training went on, with the possibility of additional reduction even after training was completed (Figure 6b). This shows that the discriminator kept learning efficiently, and training efficiency could be further enhanced by a small increase in the learning rate. Overall, examination of both networks’ loss patterns shows that the training procedure was comparatively balanced. The model successfully used tumor masking information to precisely identify regions that needed HER2 IHC staining, even in situations where 1:1 correspondence between image pairs was challenging to achieve.

The ConvNeXt-Base model outperformed other pretrained models like ViT-Base and EfficientNet-B2 in the HER2 grade classification experiment, achieving the highest accuracy of 0.9036 and F1-score of 0.8888. A wide receptive field and hierarchical feature representation are useful for identifying subtle variations in staining intensity, as demonstrated by the comparison of models of different sizes. ConvNeXt-Base correctly classified the majority of samples overall, with exceptionally high accuracy in three classes (0, 1+, and 3+), according to an analysis of the confusion matrix. This shows that the model performs evenly across several classes, indicating that it can make reliable predictions without favoring any one class over another. ConvNeXt-Base’s balanced predictive performance suggests that it can support trustworthy decisions in real-world applications. The larger models (ConvNeXt-Base and ViT-Base) achieved the best performance thanks to their expressive capacity to capture subtle staining variations. In contrast, mid-sized models such as Inception-V3 and ResNet-50V2 did not achieve comparable performance despite having more parameters than the lightweight models, suggesting that the larger models effectively overcame the limitations observed in mid-sized architectures.

However, there were some misclassifications between adjacent intensity levels, particularly between 1+ and 2+, which may represent the traits of borderline cases that are difficult even for pathologists to identify. Therefore, even though the proposed model shows good accuracy in classes with clear staining differences, it implies that more work needs to be done to distinguish fine-grained intensity variations.

### 5.2. Comparison Results Among Different Methods

The results of the qualitative evaluation demonstrated that, in comparison to other models, the proposed digital HER2 IHC staining model maintains uniform contrast and consistent staining quality. Specifically, staining intensity differences within individual images were reduced, indicating that the model achieved highly consistent generation quality for input images. Additionally, visual distortions such as general yellowish discoloration were avoided by reproducing background colors that were similar to the original. This is thought to effectively complement the issue of nonspecific region staining, a limitation of unsupervised learning. These features show that the proposed model outperforms current techniques in terms of the quality of staining image generation.

Furthermore, the proposed digital HER2 IHC staining model confirmed superior performance compared to existing models through quantitative comparison. Specifically, when compared to the DCLGAN model, the FID and KID scores decreased by 27.2% and 39.5%, respectively, and the soft accuracy increased by 2.57%. Although macro-averaged F1-score for the proposed model was marginally lower than that of DCLGAN (0.270 vs. 0.284), the proposed model’s marginal advantage in overall accuracy stems primarily from grade “2+” (34.5% of the test set), for which the proposed model achieved higher sensitivity (DCLGAN 12.1%, Ours 15.7%). In contrast, DCLGAN achieved higher sensitivity for grades “0” (1.9% of the test set) and “3+” (63.5% of the test set) (DCLGAN 28.6%, Ours 4.1%; and DCLGAN 96.3%, Ours 95.3%, respectively). Because macro-averaged F1-score weights all four grades equally regardless of sample size, and DCLGAN outperformed the proposed model in two of the four grades, its macro-F1 is marginally higher despite the proposed model’s slightly higher overall (support-weighted) accuracy.

The ablation study (Table 4) confirms that each proposed component contributes to improving the visual quality and classification consistency of the generated staining, though not uniformly across all metrics. While the tumor mask alone slightly increased FID, its combination with the mask loss led to a consistent reduction in both FID and KID, and the subsequent addition of the MS-SSIM loss and the modified identity loss further improved performance, with the full model achieving the lowest FID and KID and the highest soft accuracy and weighted F1-score among all configurations. Notably, macro-averaged F1-score followed a different trend: the tumor mask alone achieved the highest macro-F1 (0.303), whereas the full model recorded the lowest (0.27). This mirrors the DCLGAN-versus-Ours comparison discussed above, where macro-F1, being sensitive to per-class performance on underrepresented grades regardless of sample size, can diverge from support-weighted metrics such as soft accuracy and weighted F1-score. Overall, these results indicate that the individual components are not independently sufficient but act synergistically for image fidelity and overall classification agreement, while the trade-off in macro-F1 suggests that further refinement may be needed to improve consistency on minority HER2 grades.

High visual similarity in terms of image structural similarity is indicated by a lower FID and KID value, which shows that the distribution of the generated HER2 IHC images is very similar to that of the original stained images. Soft accuracy, on the other hand, indicates how closely the generated images match the originals according to pathologists’ evaluation criteria, demonstrating the model’s dependable performance from the consistency of staining patterns across grades. These findings suggest that the proposed model has the potential to digitally replace the conventional chemical staining procedure by producing HER2 IHC-stained images that are both aesthetically pleasing and reliable image generation. As a result, it should significantly improve the effectiveness of the pathological diagnostic process and reduce the experimental variability associated with staining techniques.

From a practical perspective, the computational complexity of the proposed method was also analyzed. The proposed method introduces additional components, such as tumor mask and structural losses, which slightly increase the training cost compared to the baseline DCLGAN. In practice, the training time increased by approximately 6% while maintaining a comparable number of parameters (DCLGAN 11.378M, Ours 11.381M). However, since the overall network architecture remains unchanged, the increase in computational complexity is marginal, and the inference cost is largely unaffected. However, the reported results are based on a single training run, and performance may vary across different runs due to the inherent variability of GAN-based models. Further evaluation with multiple runs and statistical analysis will be conducted in future work to improve the reliability of the results.

## 6. Conclusions

The inherent drawbacks of histological staining and the issue of general yellowish discoloration of nonspecific regions that arise in unsupervised learning were overcome in this study. We created a modified identity loss that differentiates between active and inactive regions by adding mask loss and SSIM loss to the DCLGAN structure.

In comparison to other models, we maintained uniform contrast and consistent staining quality while optimizing each model’s performance. By differentiating between background and tumor regions within images, the proposed model solved the nonspecific region problem. Quantitative results showed that staining similar to standard histological HER2 IHC staining was used, confirming visual quality through FID and consistency of staining patterns across grades through soft accuracy. However, these metrics serve only as proxy measures and do not replace expert pathological assessment. Also, we need clinical validation on a larger scale for generalized performance evaluation. Future work will create a model that fully executes DS for multiple IHC staining types in addition to HER2 IHC staining. It is anticipated that a multi-IHC staining model that learns tumor and background information based on this study’s methodology will implement precise DS while effectively making use of enormous volumes of unaligned data. In addition, recent advances such as diffusion-based models [60] and large pretrained models [61] offer more stable training and higher-fidelity results, but typically rely on large paired datasets and substantial computational resources, which are less suitable for the small, unaligned data setting addressed here. In contrast, the proposed contrastive-learning-based method operates on a small amount of unaligned data. From a modeling perspective, we will explore integrating these advances into unsupervised digital staining.

## Figures and Tables

**Table 1:** Hyperparameters and experimental settings for the digital HER2 IHC staining models. All models trained using identical settings.

Item	Setting
Input image size	256 × 256
Batch size	4
Total epochs	30
Initial learning rate	2.0 × 10^−4^
Learning rate schedule	Constant for first 10 epochs, then linear decay to 0 over the remaining 20 epochs
Optimizer	Adam (β_1_ = 0.5, β_2_ = 0.999)
GAN loss	LSGAN
Generator backbone	9 ResNet residual blocks with anti-aliased down-/up-sampling
Input normalization	(0.5, 0.5, 0.5)
Network normalization	Instance normalization
Data augmentation	None
GPUs/Framework	4 × NVIDIA TITAN Xp/PyTorch

**Table 3:** Quantitative evaluation results of digital HER2 IHC staining models. Visual quality assessed using FID, KID. Consistency of staining patterns evaluated using soft accuracy, weighted F1-score, and macro-averaged F1-score. Arrows indicate the direction of better performance (↑: higher is better; ↓: lower is better).

Model	FID (↓)	KID (×100, ↓)	Soft Accuracy (↑)	Weighted-F1 (↑)	Macro-F1 (↑)
CycleGAN	99.1	6.56 ± 0.66	57.33	0.445	0.221
CUT	65.7	3.48 ± 0.55	76.63	0.533	0.252
DCLGAN	56.6	2.91 ± 0.5	78.71	0.591	0.284
Ours	41.2	1.76 ± 0.49	80.73	0.593	0.27

**Table 4:** Ablation study evaluating the individual contribution of each proposed component (tumor mask, mask loss, MS-SSIM loss, and modified identity loss) to digital HER2 IHC staining quality, assessed using FID, KID, soft accuracy, weighted F1-score, and macro-averaged F1-score. Arrows indicate the direction of better performance (↑: higher is better; ↓: lower is better).

Model	FID (↓)	KID (×100, ↓)	Soft Accuracy (↑)	Weighted-F1 (↑)	**Macro-F1 (↑)**
DCLGAN (baseline)	56.6	2.91 ± 0.5	78.71	0.591	0.284
w/mask	58.73	2.88 ± 0.51	79.67	0.561	0.303
w/mask + mask loss	57.19	2.78 ± 0.47	77.73	0.571	0.284
w/mask + mask loss + ssim loss	52.21	2.31 ± 0.4	77	0.586	0.28
w/mask + mask loss + ssim loss + modified identity loss (Ours)	41.2	1.76 ± 0.49	80.73	0.593	0.27

## Data Availability

The data presented in this study are publicly available. These data were derived from the following resources available in the public domain: the Breast Cancer Semantic Segmentation (BCSS) dataset [55] (https://bcsegmentation.grand-challenge.org/, accessed on 27 January 2026); the AutomatiC Registration of Breast cancer tissue (ACROBAT) dataset [44] (https://acrobat.grand-challenge.org/, accessed on 27 January 2026); and the Breast Cancer Immunohistochemical (BCI) dataset [41] (https://bci.grand-challenge.org/, accessed on 27 January 2026).

## References

[1] Siegel R.L., Kratzer T.B., Giaquinto A.N., Sung H., Jemal A. (2025). Cancer Statistics, 2025. CA.

[2] Giaquinto A.N., Sung H., Newman L.A., Freedman R.A., Smith R.A., Star J., Jemal A., Siegel R.L. (2024). Breast Cancer Statistics 2024. CA Cancer J. Clin..

[3] Marianne N., Ruth T.-J. (2011). Human Diseases.

[4] Chen J.-M., Li Y., Xu J., Gong L., Wang L.-W., Liu W.-L., Liu J. (2017). Computer-Aided Prognosis on Breast Cancer with Hematoxylin and Eosin Histopathology Images: A Review. Tumor Biol..

[5] Chan J.K.C. (2014). The Wonderful Colors of the Hematoxylin–Eosin Stain in Diagnostic Surgical Pathology. Int. J. Surg. Pathol..

[6] Titford M. (2005). The Long History of Hematoxylin. Biotech. Histochem..

[7] Guan X., Zhang Z., Wang Y., Li Y., Zhang Y. (2025). Supervised Information Mining from Weakly Paired Images for Breast IHC Virtual Staining. IEEE Trans. Med. Imaging.

[8] Lin J.-R., Chen Y.-A., Campton D., Cooper J., Coy S., Yapp C., Tefft J.B., McCarty E., Ligon K.L., Rodig S.J. (2023). High-Plex Immunofluorescence Imaging and Traditional Histology of the Same Tissue Section for Discovering Image-Based Biomarkers. Nat. Cancer.

[9] Matsuno R.K., Sherman M.E., Visvanathan K., Goodman M.T., Hernandez B.Y., Lynch C.F., Ioffe O.B., Horio D., Platz C., Altekruse S.F. (2013). Agreement for Tumor Grade of Ovarian Carcinoma: Analysis of Archival Tissues from the Surveillance, Epidemiology, and End Results Residual Tissue Repository. Cancer Causes Control.

[10] Köbel M., Bak J., Bertelsen B.I., Carpen O., Grove A., Hansen E.S., Levin Jakobsen A.-M., Lidang M., Måsbäck A., Tolf A. (2014). Ovarian Carcinoma Histotype Determination Is Highly Reproducible, and Is Improved through the Use of Immunohistochemistry. Histopathology.

[11] Barnard M.E., Pyden A., Rice M.S., Linares M., Tworoger S.S., Howitt B.E., Meserve E.E., Hecht J.L. (2018). Inter-Pathologist and Pathology Report Agreement for Ovarian Tumor Characteristics in the Nurses’ Health Studies. Gynecol. Oncol..

[12] Breen J., Allen K., Zucker K., Adusumilli P., Scarsbrook A., Hall G., Orsi N.M., Ravikumar N. (2023). Artificial Intelligence in Ovarian Cancer Histopathology: A Systematic Review. npj Precis. Oncol..

[13] De Matos L.L., Trufelli D.C., De Matos M.G.L., Da Silva Pinhal M.A. (2010). Immunohistochemistry as an Important Tool in Biomarkers Detection and Clinical Practice. Biomark. Insights.

[14] Pati P., Karkampouna S., Bonollo F., Compérat E., Radić M., Spahn M., Martinelli A., Wartenberg M., Kruithof-de Julio M., Rapsomaniki M. (2024). Accelerating Histopathology Workflows with Generative AI-Based Virtually Multiplexed Tumour Profiling. Nat. Mach. Intell..

[15] Haffner I., Schierle K., Raimúndez E., Geier B., Maier D., Hasenauer J., Luber B., Walch A., Kolbe K., Riera Knorrenschild J. (2021). HER2 Expression, Test Deviations, and Their Impact on Survival in Metastatic Gastric Cancer: Results From the Prospective Multicenter VARIANZ Study. J. Clin. Oncol..

[16] Horr C., Buechler S.A. (2021). Breast Cancer Consensus Subtypes: A System for Subtyping Breast Cancer Tumors Based on Gene Expression. npj Breast Cancer.

[17] Jeon T., Kim A., Kim C. (2020). Automated Immunohistochemical Assessment Ability to Evaluate Estrogen and Progesterone Receptor Status Compared with Quantitative Reverse Transcription-Polymerase Chain Reaction in Breast Carcinoma Patients. J. Pathol. Transl. Med..

[18] Godoy-Ortiz A., Sanchez-Muñoz A., Chica Parrado M.R., Álvarez M., Ribelles N., Rueda Dominguez A., Alba E. (2019). Deciphering HER2 Breast Cancer Disease: Biological and Clinical Implications. Front. Oncol..

[19] Miglietta F., Griguolo G., Bottosso M., Giarratano T., Lo Mele M., Fassan M., Cacciatore M., Genovesi E., De Bartolo D., Vernaci G. (2021). Evolution of HER2-Low Expression from Primary to Recurrent Breast Cancer. npj Breast Cancer.

[20] Wolff A.C., Somerfield M.R., Dowsett M., Hammond M.E.H., Hayes D.F., McShane L.M., Saphner T.J., Spears P.A., Allison K.H. (2023). Human Epidermal Growth Factor Receptor 2 Testing in Breast Cancer: ASCO–College of American Pathologists Guideline Update. J. Clin. Oncol..

[21] Li F., Hu Z., Chen W., Kak A., Greenspan H., Madabhushi A., Mousavi P., Salcudean S., Duncan J., Syeda-Mahmood T., Taylor R. (2023). Adaptive Supervised PatchNCE Loss for Learning H&E-to-IHC Stain Translation with Inconsistent Groundtruth Image Pairs. Proceedings of the Medical Image Computing and Computer Assisted Intervention—MICCAI 2023.

[22] Goodfellow I., Pouget-Abadie J., Mirza M., Xu B., Warde-Farley D., Ozair S., Courville A., Bengio Y. (2020). Generative Adversarial Networks. Commun. ACM.

[23] Kim D.-B., Lee J.-H. (2025). Digital Staining Algorithm for Multi-Domain Transformation of Unstained Images. IEEE Access.

[24] Foroughi pour A., White B.S., Park J., Sheridan T.B., Chuang J.H. (2022). Deep Learning Features Encode Interpretable Morphologies within Histological Images. Sci. Rep..

[25] Liu Y., Li X., Zheng A., Zhu X., Liu S., Hu M., Luo Q., Liao H., Liu M., He Y. (2020). Predict Ki-67 Positive Cells in H&E-Stained Images Using Deep Learning Independently From IHC-Stained Images. Front. Mol. Biosci..

[26] Isola P., Zhu J.-Y., Zhou T., Efros A.A. Image-to-Image Translation with Conditional Adversarial Networks. Proceedings of the 2017 IEEE Conference on Computer Vision and Pattern Recognition (CVPR).

[27] Rivenson Y., Wang H., Wei Z., de Haan K., Zhang Y., Wu Y., Günaydın H., Zuckerman J.E., Chong T., Sisk A.E. (2019). Virtual Histological Staining of Unlabelled Tissue-Autofluorescence Images via Deep Learning. Nat. Biomed. Eng..

[28] Wang Y., Guan N., Li J., Wang X. (2024). A Virtual Staining Method Based on Self-Supervised GAN for Fourier Ptychographic Microscopy Colorful Imaging. Appl. Sci..

[29] Chae H., Kim J., Jeon J., Lee K., Lee K.C., Choi J.U., Kang S., Choi S., Bang G., Lee J.H. (2024). Restoring H&E Stain in Faded Slides via Phase-to-Color Virtual Staining in near-Infrared. APL Photonics.

[30] Rivenson Y., Liu T., Wei Z., Zhang Y., de Haan K., Ozcan A. (2019). PhaseStain: The Digital Staining of Label-Free Quantitative Phase Microscopy Images Using Deep Learning. Light Sci. Appl..

[31] Zhu J.-Y., Park T., Isola P., Efros A.A. Unpaired Image-To-Image Translation Using Cycle-Consistent Adversarial Networks. Proceedings of the 2017 IEEE International Conference on Computer Vision (ICCV).

[32] Park T., Efros A.A., Zhang R., Zhu J.-Y., Vedaldi A., Bischof H., Brox T., Frahm J.-M. (2020). Contrastive Learning for Unpaired Image-to-Image Translation. Proceedings of the Computer Vision—ECCV 2020.

[33] Han J., Shoeiby M., Petersson L., Armin M.A. Dual Contrastive Learning for Unsupervised Image-to-Image Translation. Proceedings of the 2021 IEEE/CVF Conference on Computer Vision and Pattern Recognition Workshops (CVPRW).

[34] de Haan K., Zhang Y., Zuckerman J.E., Liu T., Sisk A.E., Diaz M.F.P., Jen K.-Y., Nobori A., Liou S., Zhang S. (2021). Deep Learning-Based Transformation of H&E Stained Tissues into Special Stains. Nat. Commun..

[35] Guan X., Wang Y., Lin Y., Li X., Zhang Y. (2024). Unsupervised Multi-Domain Progressive Stain Transfer Guided by Style Encoding Dictionary. IEEE Trans. Image Process..

[36] Liu S., Zhang B., Liu Y., Han A., Shi H., Guan T., He Y. (2021). Unpaired Stain Transfer Using Pathology-Consistent Constrained Generative Adversarial Networks. IEEE Trans. Med. Imaging.

[37] Bouteldja N., Klinkhammer B.M., Schlaich T., Boor P., Merhof D. (2022). Improving Unsupervised Stain-to-Stain Translation Using Self-Supervision and Meta-Learning. J. Pathol. Inform..

[38] Li Y., Guan X., Wang Y., Zhang Y., Linguraru M.G., Dou Q., Feragen A., Giannarou S., Glocker B., Lekadir K., Schnabel J.A. (2024). Exploiting Supervision Information in Weakly Paired Images for IHC Virtual Staining. Proceedings of the Medical Image Computing and Computer Assisted Intervention—MICCAI 2024.

[39] Wang S., Zhang Z., Yan H., Xu M., Wang G. Mix-Domain Contrastive Learning For Unpaired H&E-to-IHC Stain Translation. Proceedings of the 2024 IEEE International Conference on Image Processing (ICIP).

[40] Wang Z., Bovik A.C., Sheikh H.R., Simoncelli E.P. (2004). Image Quality Assessment: From Error Visibility to Structural Similarity. IEEE Trans. Image Process..

[41] Liu S., Zhu C., Xu F., Jia X., Shi Z., Jin M. BCI: Breast Cancer Immunohistochemical Image Generation through Pyramid Pix2pix. Proceedings of the 2022 IEEE/CVF Conference on Computer Vision and Pattern Recognition Workshops (CVPRW).

[42] Heusel M., Ramsauer H., Unterthiner T., Nessler B., Hochreiter S. (2017). GANs Trained by a Two Time-Scale Update Rule Converge to a Local Nash Equilibrium. Proceedings of the 31st International Conference on Neural Information Processing Systems.

[43] Asaf M.Z., Rao B., Akram M.U., Khawaja S.G., Khan S., Truong T.M., Sekhon P., Khan I.J., Abbasi M.S. (2024). Dual Contrastive Learning Based Image-to-Image Translation of Unstained Skin Tissue into Virtually Stained H&E Images. Sci. Rep..

[44] Weitz P., Valkonen M., Solorzano L., Carr C., Kartasalo K., Boissin C., Koivukoski S., Kuusela A., Rasic D., Feng Y. (2024). The ACROBAT 2022 Challenge: Automatic Registration of Breast Cancer Tissue. Med. Image Anal..

[45] Mao X., Li Q., Xie H., Lau R.Y.K., Wang Z., Smolley S.P. Least Squares Generative Adversarial Networks. Proceedings of the 2017 IEEE International Conference on Computer Vision (ICCV).

[46] Gutmann M., Hyvärinen A. Noise-Contrastive Estimation: A New Estimation Principle for Unnormalized Statistical Models. Proceedings of the Thirteenth International Conference on Artificial Intelligence and Statistics.

[47] Liu Y., Kang X., Matsumoto K., Zhou J. (2025). VBCSNet: A Hybrid Attention-Based Multimodal Framework with Structured Self-Attention for Sentiment Classification. Chin. J. Inf. Fusion.

[48] Myakala P.K. (2025). Scaling AI with Limited Labeled Data: A Self-Supervised Learning Approach. ICCK Trans. Emerg. Top. Artif. Intell..

[49] van den Oord A., Li Y., Vinyals O. (2018). Representation Learning with Contrastive Predictive Coding. arXiv.

[50] Ounissi M., Sarbout I., Hugot J.-P., Martinez-Vinson C., Berrebi D., Racoceanu D. (2025). Scalable, Trustworthy Generative Model for Virtual Multi-Staining from H&E Whole Slide Images. PLoS Comput. Biol..

[51] Klöckner P., Teixeira J., Montezuma D., Fraga J., Horlings H.M., Cardoso J.S., Oliveira S.P. (2025). H&E to IHC Virtual Staining Methods in Breast Cancer: An Overview and Benchmarking. npj Digit. Med..

[52] Wang Z., Simoncelli E.P., Bovik A.C. Multiscale Structural Similarity for Image Quality Assessment. Proceedings of the The Thrity-Seventh Asilomar Conference on Signals, Systems & Computers.

[53] He K., Zhang X., Ren S., Sun J. Deep Residual Learning for Image Recognition. Proceedings of the 2016 IEEE Conference on Computer Vision and Pattern Recognition (CVPR).

[54] Ronneberger O., Fischer P., Brox T., Navab N., Hornegger J., Wells W.M., Frangi A.F. (2015). U-Net: Convolutional Networks for Biomedical Image Segmentation. Proceedings of the Medical Image Computing and Computer-Assisted Intervention—MICCAI 2015.

[55] Amgad M., Elfandy H., Hussein H., Atteya L.A., Elsebaie M.A.T., Abo Elnasr L.S., Sakr R.A., Salem H.S.E., Ismail A.F., Saad A.M. (2019). Structured Crowdsourcing Enables Convolutional Segmentation of Histology Images. Bioinformatics.

[56] Ayana G., Lee E., Choe S. (2024). Vision Transformers for Breast Cancer Human Epidermal Growth Factor Receptor 2 Expression Staging without Immunohistochemical Staining. Am. J. Pathol..

[57] Kingma D., Ba J. (2014). Adam: A Method for Stochastic Optimization. arXiv.

[58] Ulyanov D., Vedaldi A., Lempitsky V. (2016). Instance Normalization: The Missing Ingredient for Fast Stylization. arXiv.

[59] Metlek S. (2024). CellSegUNet: An Improved Deep Segmentation Model for the Cell Segmentation Based on UNet++ and Residual UNet Models. Neural Comput. Appl..

[60] He Y., Liu Z., Qi M., Ding S., Zhang P., Song F., Ma C., Wu H., Cai R., Feng Y. (2024). PST-Diff: Achieving High-Consistency Stain Transfer by Diffusion Models with Pathological and Structural Constraints. IEEE Trans. Med. Imaging.

[61] Chen R.J., Ding T., Lu M.Y., Williamson D.F.K., Jaume G., Song A.H., Chen B., Zhang A., Shao D., Shaban M. (2024). Towards a General-Purpose Foundation Model for Computational Pathology. Nat. Med..

